# A large colonial choanoflagellate from Mono Lake harbors live bacteria

**DOI:** 10.1128/mbio.01623-24

**Published:** 2024-08-14

**Authors:** K. H. Hake, P. T. West, K. McDonald, D. Laundon, J. Reyes-Rivera, A. Garcia De Las Bayonas, C. Feng, P. Burkhardt, D. J. Richter, J. F. Banfield, N. King

**Affiliations:** 1 Howard Hughes Medical Institute and Department of Molecular and Cell Biology, University of California, Berkeley, California, USA; 2 Department of Environmental Science, Policy, & Management, University of California, Berkeley, California, USA; 3 Electron Microscopy Laboratory, University of California, Berkeley, California, USA; 4 Marine Biological Association of the United Kingdom, Plymouth, United Kingdom; 5 Michael Sars Centre, University of Bergen, Bergen, Norway; 6 Institut de Biologia Evolutiva (CSIC-Universitat Pompeu Fabra), Barcelona, Spain; Harvard University, Cambridge, Massachusetts, USA

**Keywords:** choanoflagellates, multicellularity, bacteria, Mono Lake, fluorescence *in situ* hybridization, evolution

## Abstract

As the closest living relatives of animals, choanoflagellates offer insights into the ancestry of animal cell physiology. Here, we report the isolation and characterization of a colonial choanoflagellate from Mono Lake, California. The choanoflagellate forms large spherical colonies that are an order of magnitude larger than those formed by the closely related choanoflagellate *Salpingoeca rosetta*. In cultures maintained in the laboratory, the lumen of the spherical colony is filled with a branched network of extracellular matrix and colonized by bacteria, including diverse Gammaproteobacteria and Alphaproteobacteria. We propose to erect *Barroeca monosierra* gen. nov., sp. nov. Hake, Burkhardt, Richter, and King to accommodate this extremophile choanoflagellate. The physical association between bacteria and *B. monosierra* in culture presents a new experimental model for investigating interactions among bacteria and eukaryotes. Future work will investigate the nature of these interactions in wild populations and the mechanisms underpinning the colonization of *B. monosierra* spheres by bacteria.

**IMPORTANCE:**

The diversity of organisms that live in the extreme environment of Mono Lake (California, USA) is limited. We sought to investigate whether the closest living relatives of animals, the choanoflagellates, exist in Mono Lake, a hypersaline, alkaline, arsenic-rich environment. We repeatedly isolated members of a new species of choanoflagellate, which we have named *Barroeca monosierra*. Characterization of *B. monosierra* revealed that it forms large spherical colonies containing diverse co-isolated bacteria, providing an opportunity to investigate mechanisms underlying physical associations between eukaryotes and bacteria.

## OBSERVATION

### A newly identified choanoflagellate species forms large spherical colonies

**Fig 1:**
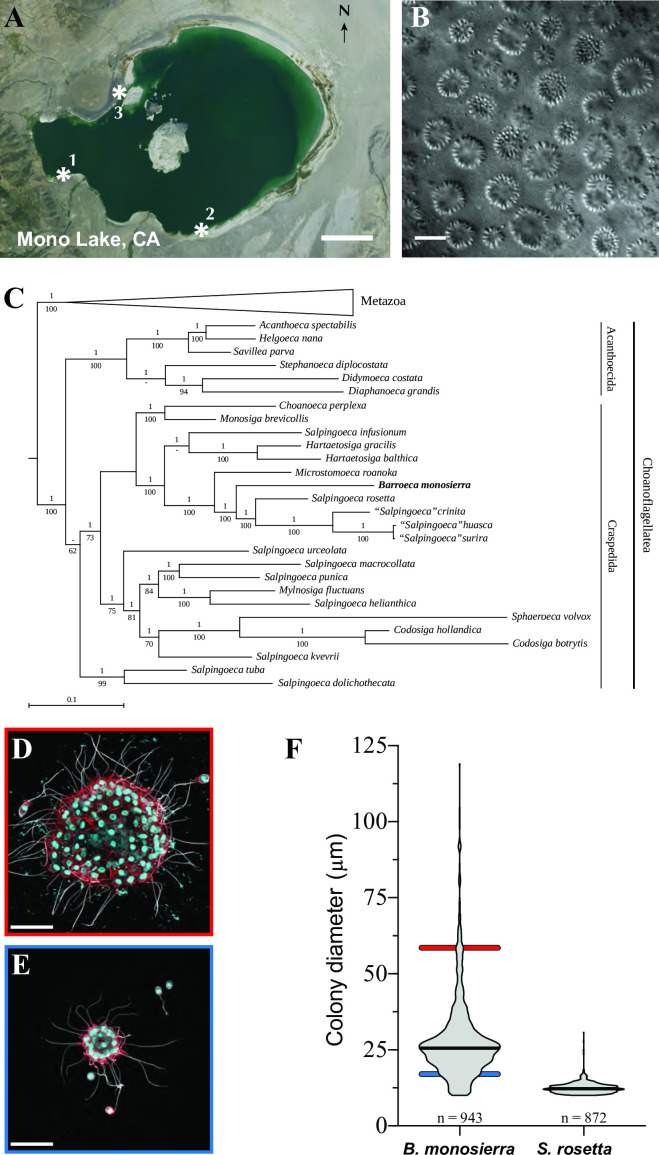
A colonial choanoflagellate isolated from Mono Lake. (**A**) Choanoflagellates were collected from three sampling sites (asterisks) near the shore of Mono Lake, California (modified from a map in the public domain, formatted as USGS Imagery Only; https://www.usgs.gov/volcanoes/long-valley-caldera) (**B**) *B. monosierra* forms large colonies (differential interference contrast image). Scale bar = 50 µm. (**C**) *B. monosierra* (shown in bold) is a craspedid choanoflagellate closely related to *S. rosetta* and *Microstomoeca roanoka*. Phylogeny based on sequences of three genes: 18S rRNA, EFL, and HSP90. Metazoa (seven species) were collapsed to save space. Bayesian posterior probabilities are indicated above each internal branch, and maximum likelihood bootstrap values are below. (A “—” value indicates a bifurcation lacking support or not present in one of the two reconstructions.) Also see Fig. S1C for further phylogenetic analyses. (**D and E**) Two colonies from the ML2.1G culture (Fig. S1, Box2) reveal the extremes of the *B. monosierra* colony size range (D, 58 µm diameter; E, 19 µm diameter; scale bar = 20 µm). In *B. monosierra* colonies, each cell is oriented with its apical flagellum (white; labeled with anti-tubulin antibody) and the apical collar of microvilli (red; stained with phalloidin) pointing out. Nuclei (cyan) were visualized with the DNA-stain Hoechst 33342. (**F**) Colonies of *B. monosierra* span from 10 µm in diameter, a size comparable to that of small *S. rosetta* colonies, to 120 µm, over an order of magnitude larger. The diameters of *B. monosierra* and *S. rosetta* colonies were plotted as a violin plot; the median is indicated as a thick black line. Diameters of the colonies in panels D and E are indicated as colored bars behind the violin plot (D, red bar; E, blue bar).

Choanoflagellates are the closest living relatives of animals and, as such, provide insights into the origin of key features of animal biology (1, 2). Over five sampling trips to Mono Lake, California (Fig. 1A; Table S1), we collected single-celled choanoflagellates and large spherical choanoflagellate colonies, many of which were seemingly hollow (Fig. 1B). In colonies and single cells, each cell bore the diagnostic collar complex observed in other choanoflagellates: an apical flagellum surrounded by a collar of microvilli (1, 2). In the spherical colonies, each cell was oriented with the basal pole of the cell body facing inwards and the apical flagellum facing out (Fig. 1B). We know of no prior reports of choanoflagellates having been isolated and cultured from any hypersaline alkaline lake, including Mono Lake.

**Fig 2:**
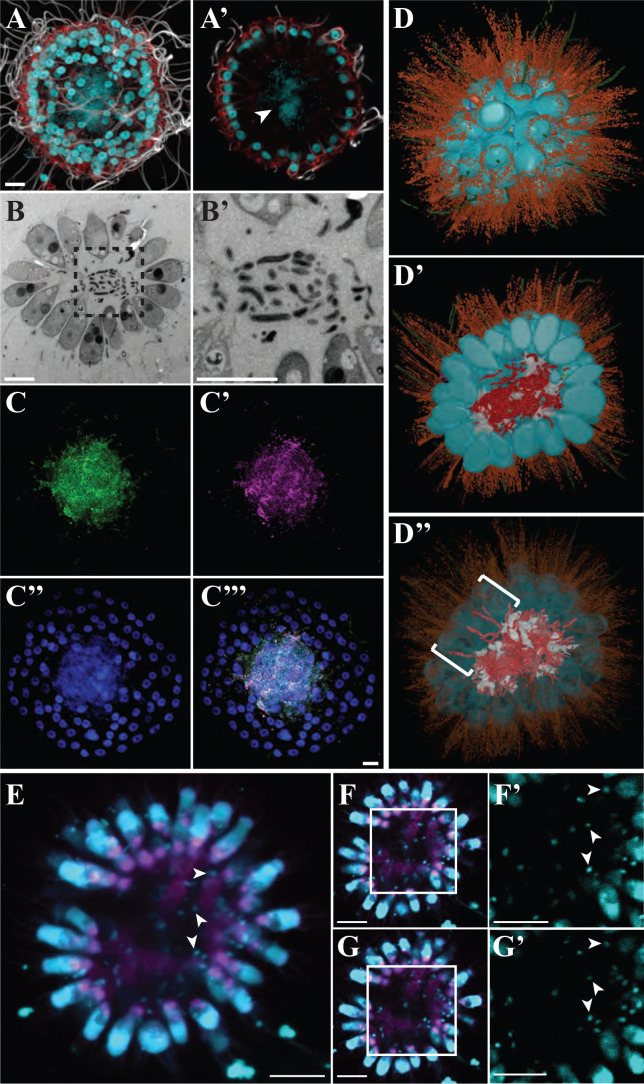
Bacteria reside in the lumina of *B. monosierra* colonies. (**A and A′**) The center of a *B. monosierra* colony from culture ML2.1G (Fig. S1, Box 2), shown as a maximum intensity projection (**A**) and optical z-section (**A′**), contains DNA (revealed by Hoechst 33342 staining; cyan). Apical flagella were labeled with anti-tubulin antibody (white); microvilli were stained with phalloidin (red). Hoechst 33342 staining (cyan) revealed the spherical choanoflagellate nuclei along the colony perimeter and an amorphous cloud of DNA sitting within the central cavity formed by the monolayer of choanoflagellate cells. (**B, B’’ and B′**) A thin section through a *B. monosierra* colony, imaged by transmission electron microscopy (TEM), revealed the presence of small cells in the central cavity. (**B′**) Inset (box from panel B) reveals that the interior cells are each surrounded by a cell wall. (**C, C’’, C’’’, and C″′′**) The small cells inside *B. monosierra* colonies (grown from cultures ML2.1E/ML2.1EC) are bacteria, as revealed by hybridization with a broad-spectrum 16S rRNA probe (C, green) and a probe targeting Gammaproteobacteria (C′, red). Choanoflagellate nuclei and bacterial nucleoids were revealed by staining with Hoechst (C″, cyan). (**C″′**) Merge of panels C, C′, and C″. Scale bar for all = 5 µm. (**D, D’ and D″**) 3D reconstruction of a 70-cell *B. monosierra* colony from transmission electron micrographs of serial ultrathin sections revealed that the bacteria are closely associated with and wrapped around the extracellular matrix (ECM) inside the colony. (**D**) Whole colony view. (**D′**) Cut-away view of colony center. False colors indicate cell bodies (cyan), microvilli (orange), flagella (green), bacteria (red), ECM (white), intercellular bridges (yellow; see also Fig. **S5**), and filopodia (purple). (**D″**) Reducing the opacity of the choanoflagellate cell renderings revealed the presence of bacteria positioned between the lateral surfaces of choanoflagellate cells (brackets; see also Fig. **S7**). (**E**) Representative *B. monosierra* colony from an environmental sample shown as an average intensity projection (planes 17–27 from 1-µm optical sections). Choanoflagellate nuclei and bacterial nucleoids (examples indicated by arrowheads) were revealed by staining with Hoechst (cyan). To allow visualization of the much smaller bacterial nucleoids, the imaging of the choanoflagellate nuclei was saturated. Concanavalin A staining (magenta) revealed the branched extracellular matrix. (**F and G**) Optical sections 19 and 24, respectively, of the *B. monosierra* colony shown in panel E. (**F′ and G′**) Higher magnification Hoechst-stained DNA from the boxed regions in F and G, highlighting the resident bacteria (examples indicated by arrowheads). Scale bars in panels E–G = 10 μm.

To study the Mono Lake choanoflagellates in greater detail, we established clonal strains from 10 independent isolates (Table S1; Fig. S1). All 10 of the original strains and additional strains generated during this study were cryopreserved for future study. Two strains (isolates ML1.1 and ML1.2) were each started from a single cell; the remaining eight were each started from a single spherical colony (Fig. S2A and B; Fig. 1B; Table S1). Despite having started as single cells, populations grown from ML1.1 and ML1.2 took on the colonial morphology observed in the other isolates after culturing in the laboratory, suggesting that this species can alternate between unicellular and colonial states. The ability to alternate between unicellular and colonial states is common among choanoflagellates (1, 3, 4).

We PCR amplified and sequenced the 18S rRNA gene from six strains (ML1.1, ML1.2, ML2.1, ML3.1, ML3.2, and ML4.3) and found them to be >99% identical (Table S1; Fig. S1C), indicating that they are all members of the same species. Phylogenetic analyses based on 18S rRNA and two protein-coding genes from isolate ML2.1 (Fig. 1C; Fig. S2C) revealed that its closest relatives are the emerging model choanoflagellate *S. rosetta* (5), other *Salpingoeca* spp. (6), and *Microstomoeca roanoka* (7, 8). The phylogenetic distance separating the Mono Lake species from its closest relatives is comparable to the distance separating other choanoflagellate genera (Text S1). Therefore, we propose erecting the genus and species name *Barroeca monosierra* Hake, Burkhardt, Richter, and King.

### Taxonomic summary

The taxonomic summary is as follows: order Craspedida Cavalier-Smith 1997 (9); family Salpingoecidae Kent (1880–1882), emend. sensu Nitsche et al. (10); genus *Barroeca* gen. nov. Hake, Burkhardt, Richter and King; uninucleated microbial eukaryote with a single, centrally positioned apical flagellum, which is surrounded by a collar of actin-supported microvilli; phagotrophic; at least some species possess an organic theca; and phylogenetically more closely related to *Barroeca monosierra* than to *Microstomoeca roanoka* or *Salpingoeca rosetta*.

#### Etymology

The genus is named for Barry S. C. Leadbeater, the author of numerous research articles and the definitive book on choanoflagellates (1) and a consistently positive influence on choanoflagellate research and researchers throughout a career spanning more than 50 years.

#### Type species

*Barroeca monosierra* Hake, Burkhardt, Richter, and King is the type species.

#### Etymology

*mono* was derived from the source locality, Mono Lake, and *sierra* for the Sierra Nevada mountain range in which Mono Lake is found.

#### Type locality

Shore of Mono Lake, California (37°58′42.7″N 119°01′52.9″W), was the location.

#### Description

The cell body is ~6–7 µm long. The apical microvillous collar is ~7.5–9.5 µm long. The apical flagellum is ~20–25 µm long. Single cells may be found attached to a substrate via a long (~30 µm) basal pedicel (Fig. S2A). Single cells may possess an organic cup-shaped theca with a distinctive ~0.75-µm outward-facing lip on its apical end (Fig. S2B). Spherical colonies can be as large as 125 µm in diameter and consist of a spheroidal arrangement of cells surrounding a hollow space containing bacteria. Adjacent cells connect via intercellular bridges, surrounded by shared plasma membrane and positioned slightly basal to cell equators. Intercellular bridges are cylindrical structures, 200–300 nm wide and 300–500 nm long, partitioned by two parallel densely osmophilic plates 175–275 nm apart.

#### Type material

The strain ML 2.1 is the one used for describing this species and is illustrated in Fig. 1 and 2.

#### Gene sequence

The partial small subunit ribosomal RNA gene sequence of strain ML2.1 has been deposited in GenBank, accession code MW838180.

### *B. monosierra* colony size and structure

Although *B. monosierra* and *S. rosetta* are closely related and both form roughly spherical colonies, their colonies differ significantly in size. *S. rosetta* colonies range from 10 to 30 µm in diameter, while *B. monosierra* forms among the largest choanoflagellate colonies thus far reported (1, 11), with a single culture containing colonies spanning from 10 to 120 µm in diameter (Fig. 1D through F). Unlike the colonies of *S. rosetta*, in which the basal poles of cells are closely apposed in the colony center (3, 11–13), cells in large *B. monosierra* colonies form a shell on the surface of a sphere. Inside the ostensibly hollow sphere, in a space analogous to a lumen, a branched network of extracellular matrix connects the basal poles of all cells (Fig. S3).

### *B. monosierra* colonies harbor live bacteria

Upon staining *B. monosierra* cultures with the DNA dye Hoechst 33342, we observed a large nucleus in each choanoflagellate cell as expected (11, 14) but were surprised to also detect Hoechst-positive material in the interior lumina of *B. monosierra* spheres (Fig. 2A and A’). Transmission electron microscopy revealed the presence of 1-µm and smaller cells with diverse morphologies in the centers of *B. monosierra* spheres (Fig. 2B and B’; Fig. S4). These observations led us to hypothesize that the centers of *B. monosierra* spheres contain bacteria.

By performing hybridization chain reaction fluorescence *in situ* hybridization (HCR-FISH [15–17]) with a broad-spectrum probe of bacterial 16S rRNA (EUB338 [18]), we confirmed that the cells in the central lumen are bacteria (Fig. 2C). A second probe that targeted 16S rRNA sequences from Gammaproteobacteria (GAM42a [19]) revealed that the majority of the bacteria in the centers of *B. monosierra* spheres are Gammaproteobacteria (Fig. 2C’). Finally, by incubating *B. monosierra* cultures with fluorescently labeled D-amino acids, which are incorporated into the cell walls of growing bacteria, we found that the bacteria in *B. monosierra* spheres are alive and growing (Fig. S5) (20).

To visualize the spatial distribution of choanoflagellate and bacterial cells in a colony, we generated a 3D reconstruction from serial sections imaged by transmission electron microscopy. The spherical colony contained 70 tightly packed choanoflagellate cells, forming an essentially continuous monolayer (Fig. 2D). As observed by immunofluorescence microscopy (Fig. 2A and A’), all cells were highly polarized and oriented with their apical flagella and collars extending away from the centroid of the sphere. Many cells were connected by fine intercellular bridges (Fig. S6) that have been previously observed in other colonial choanoflagellates, including *S. rosetta* (3, 11).

The 3D reconstruction also revealed at least 200 bacterial cells in the center of the colony (Fig. 2D’ and D”), some of which were physically associated with and wrapped around the choanoflagellate extracellular matrix (Fig. S3 and S7). A central ECM is also found in *S. rosetta* rosettes (11–13), although without the branching observed in *B. monosierra* (Fig. 2D; Fig. S7). A small number of bacterial cells were observed between the lateral surfaces of choanoflagellate cells, although it was not possible to determine whether they were entering or exiting the colony (Fig. 2D”; Fig. S8).

Freshly collected *B. monosierra* colonies that had not been cultured in the laboratory were also found to contain punctate Hoechst-positive material (Fig. 2E through G; Supplemental Notes) that we infer to belong to bacteria. Although the abundance of bacteria was lower in the freshly collected uncultured *B. monosierra* colonies relative to that of colonies from laboratory cultures, the bacteria showed similar associations with the branched ECM of the *B. monosierra* lumen (Fig. 2E). These observations imply that *B. monosierra* cultures provide a plausible system for investigating the mechanisms underlying interactions between *B. monosierra* and bacteria in nature.

To test the permeability of the colony shell to particles in the environment, we incubated *B. monosierra* cultures with bacteria-sized bovine serum albumin-coated latex microspheres (0.2 µm and 1 µm). The colonies failed to incorporate the microspheres into their centers, suggesting that bacteria from the water column (i.e., planktonic bacteria) may not be capable of passively accessing the central lumen of *B. monosierra* spheres (Fig. S9). However, some may enter through active processes.

**Fig 3:**
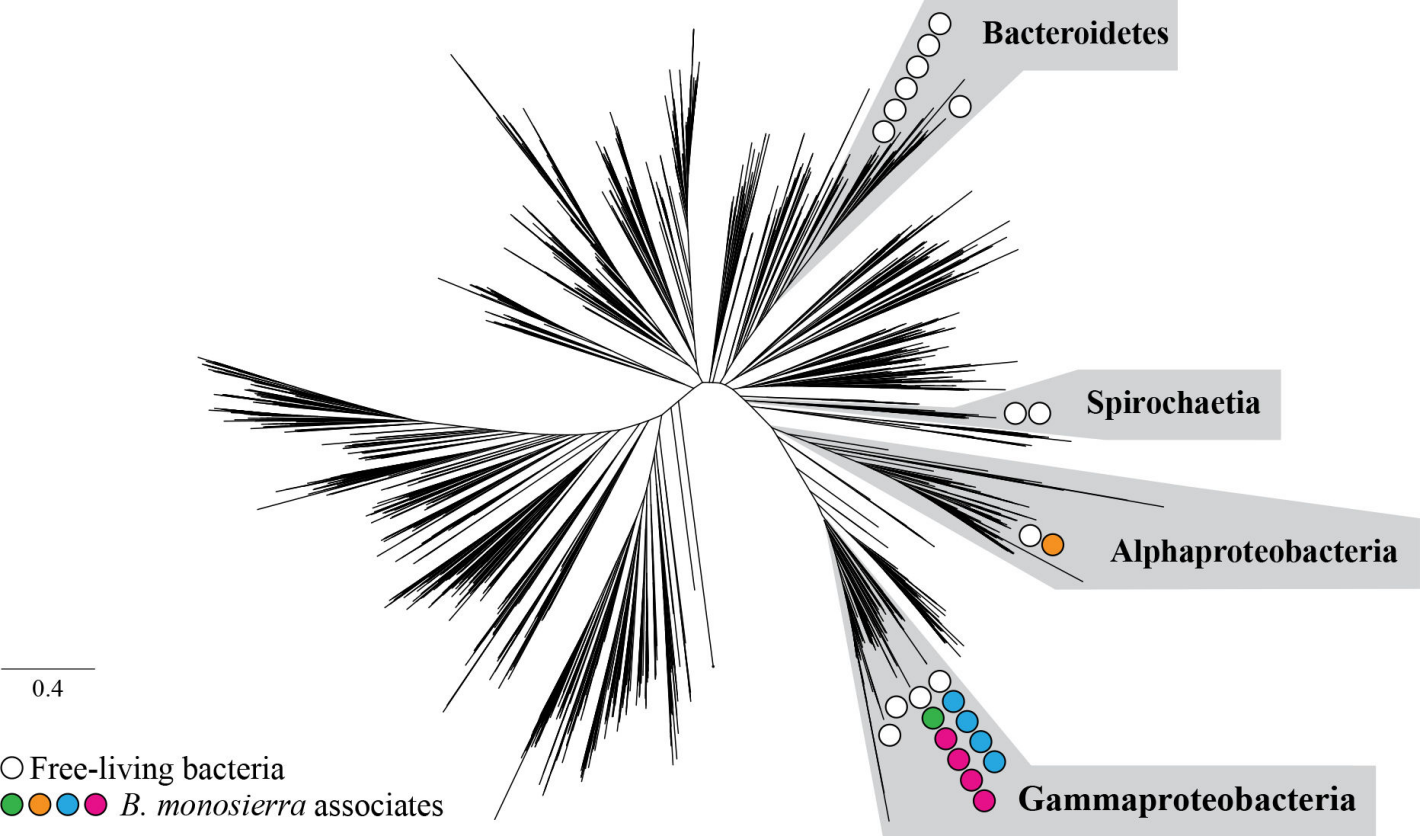
Gamma- and Alphaproteobacterial phylotypes associated with *B. monosierra*. Unrooted phylogenetic tree based on 16 concatenated ribosomal protein sequences representing bacterial diversity from reference 22, illustrated to indicate the phylogenetic placement of Mono Lake bacterial phylotypes co-cultured with *B. monosierra*. Scale bar represents the average number of substitutions per site. The bacteria belonged to four major classes: Spirochaetia, Alphaproteobacteria, Gammaproteobacteria, and Bacteroidetes; however, the bacteria found associated with *B. monosierra* colonies came only from Alphaproteobacteria and Gammaproteobacteria. Circles represent the phylogenetic placement of environmental bacteria (white) and choanoflagellate-associated bacteria (*Oceanospirillaceae* sp., magenta; *Saccharospirillaceae* sp., green; *Ectothiorhodospiraceae* sp., blue; *Roseinatronobacter* sp., orange). See also Fig. S10 and S11. The tree data file is available at https://doi.org/10.6084/m9.figshare.14474214.

After sequencing and analyzing the metagenomes of choanoflagellate-enriched and bacteria-enriched fractions of co-cultures (strains ML2.1E and ML2.1G; Fig. S1; Table S2) containing *B. monosierra* and co-isolated Mono Lake bacteria, we designed HCR-FISH probes for 16S rRNA sequences derived from all 22 phylotypes of bacteria that were detected in choanoflagellate-enriched fractions (Tables S3 and S4; see Text S1 for methods, a description of probe design, and tests of probe specificity). Phylogenetic analysis of highly conserved ribosomal proteins and 16S rRNA sequences revealed that the bacteria maintained in culture with *B. monosierra* represent a subset of the bacterial families, orders, and phyla previously detected in metagenomic analyses of Mono Lake (21) (Fig. 3; Fig. S10). For example, members of order Oceanospiralles and family Ectothiorhodospiraceae were detected in prior metagenomic surveys of Mono Lake (21) and, in this study, in the lumen of *B. monosierra* (Fig. S11A and B; Fig. S13). In contrast, although class Spirochaetia and phylum Bacteroidetes were detected as abundant members of the Mono Lake microbiota in our analyses (Fig. 2E; Fig. S10; Table S3) and those of Edwardson and Hollibaugh (21), they were not detected in the lumen of *B. monosierra* (Fig. 3).

Although most bacteria detected in the lumina of *B. monosierra* spheres were members of class Gammaproteobacteria, they exhibited an array of morphologies, from long and filamentous to rod shaped (Fig. S4 and S12). Intriguingly, except for OceaML3 (family Oceanospirillaceae), which was exclusively detected inside *B. monosierra* colonies of ML2.1E (Fig. S13), all other bacterial phylotypes identified in this study were detected both inside the colonies and in the water column. Only one bacterial phylotype tested, OceaML1 (family Oceanospirillaceae), was found in all *B. monosierra* colonies (Fig. S14A). The other most frequently observed bacteria were SaccML (93.3% of colonies; family Saccharospirillaceae), EctoML3 (91.8% of colonies; family Ectothiorhodospiraceae), and EctoML1 (82.4% of colonies; family Ectothiorhodospiraceae; Fig. S14A). The most common resident of *B. monosierra* colonies, OceaML1, was also the most abundant, representing, on average, 66.4% of the total bacterial load per sphere (Fig. S14B). The only Alphaproteobacterium detected in *B. monosierra* spheres, RoseML, was the least abundant and least frequently detected phylotype of those for which FISH probes were tested. The current study did not investigate the extent to which these different bacterial phylotypes are represented in the spheres of natural populations of *B. monosierra*. Nonetheless, the establishment of *B. monosierra* cultures that maintain physical associations with diverse bacteria provides a new model system in which to investigate mechanisms underlying interactions among eukaryotes and bacteria.

### Conclusion

Interactions with bacteria are essential for choanoflagellate nutrition and life history. Bacteria are the primary food source for choanoflagellates (1, 23), and the choanoflagellate *S. rosetta* initiates multicellular development and mating in response to different secreted bacterial cues (24–27). Here, we report the isolation and characterization of a new choanoflagellate species, *B. monosierra*, that forms large colonies containing bacteria. To our knowledge, this is the first report of such an interaction between choanoflagellates and bacteria.

*B. monosierra* and its associated bacteria provide a unique opportunity to characterize the interactions among a single choanoflagellate species and its microbial consorts. We note that while fresh isolates of *B. monosierra* harbor bacteria in their lumen (Fig. 2E through G), the specific host-microbe associations reported here were characterized in laboratory cultures of *B. monosierra*. Further studies of wild-caught populations of *B. monosierra*, freshly collected from Mono Lake, will help illuminate whether the bacterial phylotypes identified here are natural residents of *B. monosierra* spheres. While we do not yet know whether the bacterial associates of cultured *B. monosierra* perfectly reflect the phylotypes of bacteria found in natural populations, we note that studies of interactions between the nematode *Caenorhabditis elegans* and the Gammaproteobacterium *Escherichia coli* have been tremendously informative about host/microbe interactions, despite the fact that the two species do not encounter each other in nature. Finally, the close phylogenetic relationship between choanoflagellate and animals suggests that the interactions among *B. monosierra* and its bacterial residents have the potential to illuminate the ancestry of and mechanisms underlying associations between animals and bacteria.

## Data Availability

GenBank accession numbers for bacterial 16S rRNA sequences are listed in Table S5. Sequences for *B. monosierra* 18S rRNA, EFL, and Hsp90 (Fig. 1E) have been assigned GenBank accession numbers MW838180, MW979373, and MW979374, respectively. 18S sequences for different *B. monosierra* strains (Table S1) have been assigned GenBank accession numbers MZ015010 to MZ015015. Raw reads from the *B. monosierra* genome sequence are available under the Genbank accession number PRJNA734368. The assembled *B. monosierra* genome, all bacterial genome sequences, and all relevant input and output data from the phylogenetic trees presented in Fig. 1C and 2E and Fig. S1 are available via FigShare (https://doi.org/10.6084/m9.figshare.14474214).

## SUPPLEMENTAL MATERIAL

File S1 mbio.01623-24-s0001.pdf Mono Lake FISH probe specificity.

Supplemental Material mbio.01623-24-s0002.xlsx Supplemental taxonomic notes, materials and methods, figures, and tables.

ASM does not own the copyrights to Supplemental Material that may be linked to, or accessed through, an article. The authors have granted ASM a non-exclusive, world-wide license to publish the Supplemental Material files. Please contact the corresponding author directly for reuse.
