# Supplementary material for: A large colonial choanoflagellate from Mono Lake harbors live bacteria
Source: mBio. 2024 Aug 14;15(9):e01623-24. doi: 10.1128/mbio.01623-24 (PMC11389367; doi:10.1128/mbio.01623-24)
Supplement: File S1 — Mono Lake FISH probe specificity. [file mbio.01623-24-s0001.pdf]

SUPPLEMENT

for

A large colonial choanoflagellate from Mono Lake harbors live bacteria

Hake, K. H.[1, 2], West, P.T.[3], McDonald, K.[4], Laundon, D.[5], Reyes-Rivera, J.[1], Garcia De Las Bayonas, A.[1], Feng, C.[1], Burkhardt, P.[5, 6], Richter, D.J.[7*], Banfield, J.F.[3*], and King, N.[1,*]

[1]Howard Hughes Medical Institute and Department of Molecular and Cell Biology, University of California, Berkeley, CA, USA

[2] Present address: Calico Life Sciences, South San Francisco, CA, USA

[3] Department of Environmental Science, Policy, & Management, University of California, Berkeley, CA, USA

[4] Electron Microscopy Laboratory, University of California, Berkeley, CA, USA

[5] Marine Biological Association of the United Kingdom, Plymouth, United Kingdom. Present address: University of Southampton.

[6] Michael Sars Centre, University of Bergen, Bergen, Norway

[7]Institut de Biologia Evolutiva (CSIC-Universitat Pompeu Fabra), Barcelona, Spain

[*]Co-corresponding authors

Contents

Text S1: Taxonomic Notes and Supplemental Materials and Methods

Figure S1: Establishment of *B. monosierra* cultures.
Figure S2: Ultrastructure and phylogeny of choanoflagellate isolates from Mono Lake.
Figure S3: *B. monosierra* rosettes contain an extracellular matrix (ECM).
Figure S4: Bacterial residents in *B. monosierra* rosettes exhibit a range of morphologies.
Figure S5: Bacteria inside *B. monosierra* rosettes are alive and growing.
Figure S6: Intercellular bridges connect cells in *B. monosierra* rosettes.
Figure S7: Bacterial residents physically associate with and wrap around the choanoflagellate ECM.
Figure S8: Bacteria are found wedged between the lateral surfaces of *B. monosierra* cells.
Figure S9: *B. monosierra* rosettes cannot be passively penetrated by sub-micron particles.
Figure S10: Phylogenetic analysis of 16S rRNA sequences.
Figure S11: Diverse bacteria are found in the lumen of *B. monosierra.*
Figure S12: Resident bacteria of *B. monosierra* exhibit filamentous and rod morphologies.
Figure S13: The bacterium OceaML3 was detected exclusively inside rosettes.
Figure S14: OceaML1 is a core member of the *B. monosierra* bacterial community.
Figure S15: Bacterial community overlap across shotgun metagenomic sequencing samples.

Table S1: Date, location, phenotype, and isolate designations for *Barroeca monosierra* isolates.
Table S2: Shotgun metagenomic sequencing project outcomes
Table S3: Nine genera of bacteria identified in *B. monosierra* cultures through two independent analyses: comparison of ribosomal proteins detected through metagenomic assembly and by 16s rRNA assembly and analysis.
Table S4: Predicted targets of HCR-FISH probes based on 16S rRNA sequences.
Table S5: Bacterial 16S rRNA – Genbank accession numbers
Table S6: Full length probes, with spacer and initiator sequences, used for HCR-FISH.
Table S7: Growth Media Recipes.
Table S8: Dereplicated bin set used for constructing the ribosomal protein tree

Supplemental Data File 1. Mono Lake FISH Probe specificity.

Text S1: Taxonomic Notes and Supplemental Materials and Methods

We propose the creation of a new genus for the Mono Lake species for the following four reasons:

1. The phylogenetic distance separating the Mono Lake species from its closest relatives (*M. roanoka* and *S. rosetta*) is equivalent to the distance between genera in other parts of the choanoflagellate tree (Fig. 1C; for example, between *C. perplexa* and *M. brevicollis*).

2. The internal branch joining the Mono Lake species together with the *S. rosetta* group is short, indicating that they probably did not diverge long after the split from *M. roanoka*. This is similar to the situation observed within the Acanthoecida (where short internal branches lead to many separate genera) and stands in contrast to other bona fide genera in Craspedida that do share a long internal branch, such as *Hartaetosiga* or *Codosiga*.

3. It would be preferable to avoid adding another species to the *Salpingoeca* genus, which is already highly paraphyletic. Adding another *Salpingoeca* would increase confusion when interpreting the relationship among species (which outweigh the disadvantages of creating a monospecific genus, of which numerous examples currently exist within Craspedida: for example, *Microstomoeca* and *Mylnosiga*). Furthermore, as the type species of *Salpingoeca* has not yet been sequenced, creating a new genus for the Mono Lake species would avoid the possibility of its needing to be transferred to another genus at a later date.

4. Choanoflagellates and animals diverged at least 600 million years ago, and the average phylogenetic distance between any two choanoflagellates is at least as great as the average phylogenetic distance between any two animal phyla [1]. Thus, the Mono Lake species is likely to have experienced at least tens of millions of years of independent evolution after separating from its closest known relatives in the tree. In fact, a recent study, which did not include the Mono Lake species, estimated the divergence of *M. roanoka* and *S. rosetta* to have occurred roughly 100 million years ago, and the divergence of *S. rosetta* from its closest relatives to have occurred roughly 38 million years ago [2].

Initial isolation of choanoflagellate *B. monosierra*

B. monosierra was originally isolated from water samples collected at Mono Lake, CA from 2012-2014 (Table S1; Fig. 1A). 30 mL of lake water was collected in a T25 cell culture flask with a vented cap (Thermo Fisher Scientific, Waltham, MA; Cat. No. 10-126-28). The flasks were placed in the dark for 2-4 weeks at 22˚C to reduce the load of photosynthetic microorganisms and allow the choanoflagellates to grow and increase in density. Flasks were visually screened for the presence of choanoflagellate cells, and the choanoflagellates were clonally isolated by two serial dilution-to-extinction steps, similar to the method described in (Fig. S1)[5]

Choanoflagellate Growth Media

Mono Lake water was collected in large volumes from the *B. monosierra* isolation locations (Table S1) and sterile-filtered to remove any bacterial contaminants that may have been introduced in transit. *B. monosierra* and its associated environmental and lumen bacteria were initially transferred into and grown in the 0.22 µm filtered Mono Lake water. The cultures were later transferred into and propagated in artificial Mono Lake water (AFML) designed to approximate the water chemistry of Mono Lake, based on assessments by Dr. Frank Nitsche

(University of Cologne) and the Los Angeles Department of Water and Power (LADWP; http://www.monobasinresearch.org/images/mbeir/dchapter3/table3b-2.pdf). AFML was prepared by adding salts and minerals (Table S7) to MilliQ water and filtering through a 0.22 µm filter without autoclaving. Calcium chloride dihydrate was added as a stock solution (1:1000). 1000x Trace elements and 1000x L1 Vitamins were added to AFML right before use as described in [6] where they were added to artificial sea water (ASW) for culturing *S. rosetta*. Mono Lake Cereal grass medium (CGM3ML) was made by infusing unenriched freshly autoclaved AFML with cereal grass pellets (5g/L) (Carolina Biological Supply, Burlington, NC; Cat. No. 132375) as previously described [7]. Davis Mingolis synthetic media (DMSM)[8] was made by adding the chemicals described here (Table S7) to MilliQ water and sterile filtering through a 0.22 µm filter without autoclaving. *S. rosetta* was propagated in unenriched sea water (32.9 g Tropic Marin sea salts to 1L water) with 5% (vol/vol) sea water complete medium [9] resulting in HN medium (250 mg/L peptone, 150 mg/L yeast extract, and 150 µl/L glycerol in unenriched sea water) as previously described [7].

Culturing conditions and establishment of ML2.1 Cultures

Clonal isolates of *B. monosierra* cultures were passaged once a week by adding 3 mL of culture to 9 mL of 10% CGM3ML diluted in AFML with added vitamins and trace minerals into a T75 vented cell-culture flask. Frozen stocks were prepared as previously described [10]. The optimal growth conditions for *B. monosierra* were tested by growing the culture in different concentrations of media previously used for other choanoflagellate species [1,7,11] as well as media used to isolate bacteria from alkaline soda lakes [8]. We found that *B. monosierra* grew best under two conditions (Fig. S1, Box 2). The first condition involved passaging 3 mL of *B. monosierra* culture in 9 mL of 2.5% DMSM diluted in AFML and resulted in the culture ML2.1E. The second involved passaging 3 mL of *B. monosierra* in 9 mL of 10% CGM3ML diluted in AFML that was treated with Gentamicin (50 µg/mL) over six weeks and then maintained in 10% CGM3ML diluted in AFML resulting in the culture ML2.1G.

Cultures ML2.1E and ML2.1G were passaged under these conditions for 4 months before being sequenced (Fig. S1, Box 3). Shortly after sequencing, ML2.1G became overpopulated with bacteria that outcompeted the choanoflagellates. We were not able to recover this culture from a freeze down. Due to the variability in growth of ML2.1E, likely due to the diversity of bacterial prey and their growth dynamics, we found it was helpful to always have two cultures growing in different media to improve the chances of having a healthy culture on hand. Therefore, we began passaging ML2.1E in not only 2.5% DMSM, but also 10% CGM3ML resulting in the culture ML2.1EC (Fig. S1, Box 4). Experiments were performed in either ML2.1E or ML2.1EC based on the quality of the cultures on the day of the experiment.

High-throughput colony size analysis

For the colony size analysis in Fig. 1F, the ML2.1G culture was used for the *B. monosierra* sample. For *S. rosetta,* the colony strain Px1 (ATCC PRA-366: https://www.atcc.org/Products/All/PRA-366.aspx), a monoxenic culture consisting of *S. rosetta* and the rosette-inducing bacterium *A. machipongonensis,* was used for analysis [12]. Rosettes were concentrated ten-fold by centrifugation at 2000xg for 5 min. Cells were stained with LysoTracker Red DND-99 (Thermo Fisher Scientific; Cat. No. L7528) at a concentration of 1 µl per 100 µl of cells. This concentration of LysoTracker selectively labels the entire cell body of the choanoflagellate and not prey bacteria.

Colonies were imaged by immediately placing a 20 µl drop of stained cells on a glass slide and gently squishing with a 24x40 coverslip (Fisher Scientific; Cat. No. 12-545D). Slides were imaged immediately using a Zeiss Axio Observer.Z1/7 Widefield microscope with a Hamamatsu Orca-Flash 4.0 LT CMOS Digital Camera (Hamamatsu Photonics, Hamamatsu City, Japan) and 20X/NA 0.8 Plan-Apochromat (Zeiss). Four 100-image tilescans with 0% image overlap were acquired resulting in 400 images per sample.

Images were analyzed on FIJI [13] and scanned for quality by hand. Any image with debris or out of focus choanoflagellate colonies was deleted. An automatic threshold was set using the intermodes method [14]. Measurements were set to collect area and Feret's diameter. Images were analyzed using the 'Analyze Particles' command with settings to exclude on edges and to include holes. Minimum rosette area and diameter were set to 35 µm and 10 µm, respectively, based on *S. rosetta* measurements of 2-cell rosettes. Total number of rosettes imaged was 943 for *B. monosierra,* and 872 for *S. rosetta.* Data were presented as a violin boxplot made using GraphPad Prism8 showing the median area or diameter (black line), and the kernel density trace (black outline) plotted symmetrically to show frequency distribution for the given measurements.

Immunofluorescence, confocal imaging, and live cell microscopy

For immunofluorescence staining of *B. monosierra* (Fig. 1D and E; Fig. 2A and A'), a round poly-L-lysine coated coverslip (BD Biosciences) was placed at the bottom of a 24 well plate. 1750 µl of *B. monosierra* culture was added to each well with a coverslip followed by 250 µl of 32% Paraformaldehyde (PFA) resulting in a final concentration of 4%PFA. The 24 well plate was spun at 1000xg for 15 minutes at room temperature to concentrate choanoflagellate colonies onto the coverslip. The fixative and culture media was replaced with PEM (100 mM PIPES-KOH, pH 6.95; 2 mM EGTA; 1 mM $MgCl_2$). Immunofluorescence was continued as previously described (13) with 50 ng/ml mouse E7 anti-β-tubulin antibody (Developmental Studies Hybridoma Bank, University of Iowa, Iowa City, IA; Cat. No. AB_2315513), 8 ng/ml Alexa Fluor Plus 488 Goat anti-Mouse IgG (H+L) secondary antibody (Thermo Fisher Scientific; Cat. No. A32723), 4 U/ml Rhodamine phalloidin (Thermo Fisher Scientific; Cat. No. R415), and 0.1mg/mL Hoechst 33342 (Thermo Fisher Scientific; Cat. No. H3570). Coverslips were mounted in Pro-Long Diamond antifade reagent (Thermo Fisher Scientific; Cat. No. P36970) and left to cure overnight before imaging.

Confocal images were acquired using a 63X/NA 1.40 Plan-Apochromatic oil immersion objective on either a Zeiss LSM 700 confocal microscope or a Zeiss LSM 880 Airyscan confocal microscope with an Airyscan detector (Carl Zeiss AG, Oberkochen, Germany). Images acquired using the Airyscan detector were processed using the automated Airyscan algorithm (Zeiss) and then reprocessed with the Airyscan threshold 0.5 units higher than the automated reported threshold.

For live epifluorescence and differential interference contrast (DIC) imaging (Fig. 1B), cultures of either *B. monosierra* or *S. rosetta* were typically concentrated tenfold, stained with a combination of dyes, and imaged similarly to the colony size analysis by placing 20 µl of cell culture on a slide and gently squishing with a 24x40 coverslip. To stain the DNA, cultures were stained ten minutes with 0.1 mg/mL Hoechst 33342 (Thermo Fisher Scientific; Cat. No. H3570). To label the ECM of *B. monosierra* (Fig. S3), fluorescein-labeled Concanavalin A (Con A) (Vector Labs, Burlingame, CA; Cat No. FL-1001), or for *S. rosetta,* fluorescein labeled Jacalin (Vector Labs; Cat No. FL-1151) was added to cultures at a concentration of 5 µg/mL for five minutes. LysoTracker DND-99 (Thermo Fisher Scientific; Cat. No. L7528) labeled the

choanoflagellate colonies as described in the colony size analysis. Cultures were imaged without washing the dyes off. Slides were imaged using a Zeiss Axio Observer.Z1/7 Widefield microscope with Hamamatsu Orca-Flash 4.0 LT CMOS Digital Camera and a 100x NA 1.40 Plan-Apochromatic oil immersion objective (Zeiss).

Labeling cultures with D-amino acids, and incubating with fluorescent beads

To label growing bacteria with fluorescently labeled D-amino acids (Fig. S5), cultures were concentrated fivefold and HADA D-amino acids were added at a concentration of 2 mM [15] and incubated for 24 hours. The cultures were imaged as described in live cell microscopy. To test rosette permeability (Fig. S8), 2 mL of ML 2.1 grown in AFML was concentrated twofold and 0.2 µm Fluospheres® (Thermo Fisher Scientific; Cat. No. F8848) and 1 µm Fluospheres® (Thermo Fisher Scientific; Cat. No. F8851) were added at a concentration of 1:100. Cultures were left with beads for 24 hours and imaged as described in live cell microscopy.

Transmission electron microscopy (TEM) and 3D reconstruction

For the TEM images (Fig. 2B, B'; Fig. S4; Fig. S6-8) we modified methods previously established for *S. rosetta* [16,17]. We first concentrated 40 mL of cultured *B. monosierra* rosettes by gentle centrifugation (200xg for 30min) resuspended in 20% BSA (Bovine Serum Albumin, Sigma) made up in artificial seawater medium, and concentrated again. Most of the supernatant was removed and the concentrated cells transferred to high-pressure freezing planchettes varying in depth between 50 and 200 µm (Wohlwend Engineering). Freezing was done in a Bal-Tec HPM-010 high-pressure freezer (Bal-Tec AG).

The frozen cells were stored in liquid nitrogen until needed, and then transferred to cryovials containing 1.5 mL of fixative consisting of 1% osmium tetroxide plus 0.1% uranyl acetate in acetone at liquid nitrogen temperature (−195°C) and processed for freeze substitution according to the method described here [18,19]. Briefly, the cryovials containing fixative and cells were transferred to a cooled metal block at −195°C (the cold block was put into an insulated container such that the vials were horizontally oriented) and shaken on an orbital shaker operating at 125 rpm. After 3 h, the block/cells had warmed to 20°C and were ready for resin infiltration.

Resin infiltration was accomplished according to the method of described here [18]. Briefly, cells were rinsed three times in pure acetone and infiltrated with Epon-Araldite resin in increasing increments of 25% over 30 min plus three changes of pure resin at 10 min each. Cells were removed from the planchettes at the beginning of the infiltration series and spun down at 6,000x g for 1 min between solution changes. The cells in pure resin were placed in between two PTFE-coated microscope slides and polymerized over 2 h in an oven set to 100°C. Images of cells were taken on an FEI Tecnai 12 electron microscope.

For the 3D reconstruction in Fig. 2D-D'', we collected images from serial sections followed the approach previously described in [17]. Briefly, the cells were cut from the thin layer of polymerized resin and remounted on blank resin blocks for sectioning. Serial sections of varying thicknesses between 70–150 nm were cut on a Reichert-Jung Ultracut E microtome and picked up on 1 x 2-mm slot grids covered with a 0.6% Formvar film. Sections were post-stained with 1% aqueous uranyl acetate for 7 min and lead citrate for 4 min. Images of sections were collected on an FEI Tecnai 12 electron microscope.

The 3D reconstruction of an entire rosette was achieved using serial ultrathin TEM sectioning (ssTEM). 176 images taken from consecutive 150 nm thick sections were compiled into a single

stack and imported into the Fiji [20] plugin TrakEM2 [21]. Stack slices were automatically aligned using default parameters with minor modifications (steps per octave scale were increased to 5 and maximal alignment error reduced to 50 px). Automatic alignments were curated and manually corrected if unsatisfactory. Cellular structures were segmented manually, and 3D reconstructed by automatically merging traced features along the z-axis. Meshes were then smoothed in TrakEM2, exported into the open-source 3D software Blender 2.77, and rendered for presentation purposes only.

Collection and imaging of environmental samples

Fresh environmental samples of *B. monosierra* were collected near Black Point parking lot at Mono Lake (Fig. 1, Table S1). ~20 mL of lake water was collected in each of multiple T25 cell culture flasks with vented caps (Thermo Fisher Scientific, Waltham, MA; Cat. No. 10-126-28). The flasks were screened under 40X magnification using an inverted lens clinical microscope (Leica DMIL) to identify those containing *B. monosierra* colonies. The flasks that contained colonies (and other environmental microbial eukaryotes and bacteria) were then transported to our UC Berkeley laboratory in a soft-sided cooler with a few ice cubes to prevent overheating of the samples.

Because the predator/prey dynamics in natural samples from Mono Lake are dynamic, it was necessary to work rapidly to avoid loss of the *B. monosierra* colonies. Within two days after collection of the samples, they were fully processed for imaging (Fig. 2 E-G). To image the ECM of *B. monosierra*, 400 µl of an environmental sample containing *B. monosierra* colonies was incubated for 15 min. at room temperature with 2 µl of a 1 mg/mL stock of fluorescein-labeled Con A (Vector Labs, Burlingame, CA; Cat No. FL-1001), yielding a final concentration of 5 µg/mL Con A. To remove excess Con A, the samples were pelleted by spinning at 2500 xg for 5 min., the supernatant was carefully removed by pipetting, and the pellet was resuspended in 400 µl filter-sterilized Mono Lake water.

To fix the samples, 400 µl of Con A-stained material was transferred to a poly-D-lysine coated black square 96-well plate (ibidi, 89626) and treated with 32 µl 16% Paraformaldehyde (PFA) resulting in a final concentration of 4%PFA. The cells were incubated for 15 min. with gentle rocking. To stain for DNA, 4 µl of 0.1mg/mL Hoechst 33342 (Thermo Fisher Scientific; Cat. No. H3570) was added to the fixed cells (final concentration of 0.1 µg/mL Hoechst) and incubated with gentle rocking for 10 minutes.

Images of the Hoechst- and Con A-stained environmental samples were captured using an inverted Nikon Eclipse Ti microscope (Nikon Instruments) with an oil-immersion objective (Apo 60x, NA 1.49, oil). A z-stack was acquired for each rosette and the images were processed for presentation in FIJI [13]. Contrast and brightness were adjusted linearly to improve observation of the choanoflagellate cells (which stain brightly with Con A and Hoechst) and their resident bacteria (which stain less brightly with Hoechst and not at all with Con A).

Genomic DNA extraction and sequencing

Colonies and free-living bacteria were separated by differential centrifugation. To enrich for large rosettes, 10 mL of dense culture was concentrated in a clinical centrifuge at 200xg for 30min. The supernatant was transferred to a clean 15 mL conical tube and set aside for bacterial enrichment. We resuspended the rosette pellet twice in 15 mL of AFML and centrifuged at 2000xg for 10 minutes to separate the choanoflagellates from free-living bacteria. The rosette pellet was transferred in residual media to a 1.5 mL tube, confirmed by microscopy

that it had enriched, and then was pelleted and frozen under liquid nitrogen. Free-living bacteria were enriched from the first supernatant by centrifuging at 500xg for 10 minutes to deplete single cell choanoflagellates and small rosettes that didn't pellet in the first spin. The supernatant was concentrated at 4000xg for 20 min. Concentrated bacteria were re-suspended in 1 mL of residual media and filtered through a 3 µm filter to remove single cell choanoflagellates. Bacteria were transferred to a 1.5 mL tube, confirmed by microscopy that there were few choanoflagellates, pelleted, and frozen under liquid nitrogen. A phenol-chloroform extraction was performed to generate gDNA. Samples were sequenced with 150 bp Illumina paired end reads to a depth ranging from 22.4 to 34.1 Gbp.

Metagenome assembly, annotation, and binning

Sequencing reads were processed with bbtools (http://jgi.doe. gov/data-and-tools/bbtools/) to remove Illumina adaptors as well as phiX Illumina trace contaminants. Reads were then quality-filtered with SICKLE (https://github.com/ najoshi/sickle). IBDA_UD [22] was used to assemble and scaffold filtered reads from each sample with default parameters. Putative eukaryotic scaffolds were filtered with EukRep [23] prior to bacterial binning. Protein coding sequences were predicted using MetaProdigal [24] on whole assembled metagenomic samples. Bacterial 16S sequences were reconstructed with EMIRGE (v. 0.61.0)[25]. The exact number of 16S rRNA sequences present in each sample could not be determined due to the co-assembly of 16S rRNAs from closely related species in shotgun metagenome samples [25]. In addition, 16S sequences frequently do not bin into genomes due to their high copy number, so they could not be tied directly to binned genomes. The choanoflagellate 18S rRNA sequence was identified with an HMM-based approach (https://github.com/christophertbrown/bioscripts), where a bacterial 16S rRNA, archaeal 16S rRNA, and eukaryotic 18s rRNA model were run concurrently and overlapping predictions were picked based on the best alignment. Genome bins were identified and refined using ggKbase (ggkbase.berkeley.edu) to manually check the GC, coverage, and phylogenetic profiles of each bin. dRep [26] was used to de-replicate genomic bins across samples. The results and analyses from each of the metagenome sequencing and assembly processes is summarized in Table S2 and the bacterial community overlap between different samples is displayed in Fig. S15.

18S rRNA sequencing of Mono Lake isolates

We amplified the 18S rRNA gene from 6 Mono Lake choanoflagellate isolates via PCR of genomic DNA with the universal eukaryotic 18S primers 1F (5' AACCTGGTTGATCCTGCCAGT 3') and 1528R (5' TGATCCTTCTGCAGGTTCACC 3')[27]. We cloned PCR products using the TOPO TA Cloning vector from Invitrogen, following the manufacturer's protocol. We next performed Sanger sequencing on multiple clones per isolate, using the T7 forward primer and the M13 reverse primer on the vector backbone, which produced between 2-9 successful clones per isolate, after removing empty vector and contaminant sequences. For each isolate, we aligned sequences using FSA version 1.15.7 [28] with the '--fast' option and then inferred a majority-rule consensus sequence from the alignment.

Phylogenetic analysis of choanoflagellate sequences

To place the newly isolated Mono Lake species in a phylogenetic context (Figs. 1C and S2), we began with the sequences used for tree reconstruction in [1], selecting only the choanoflagellate species and 7 representative animals (Porifera: *Amphimedon queenslandica*, Ctenophora: *Mnemiopsis leidyi*, Placozoa: *Trichoplax adhaerens*, Cnidaria: *Nematostella vectensis*, Ecdysozoa: *Daphnia pulex*, Deuterostomia: *Mus musculus*, Lophotrochozoa: *Capitella teleta*).

We then added the 18S rRNA sequences of three species closely related to *S. rosetta*: *S. crinita*, *S. huasca* and *S. surira* [29]. We built one tree (Fig. S2) incorporating the six 18S sequences we obtained from colonial Mono Lake cultures via PCR (Table S1). Next, we searched the genome sequence for the ML 2.1 isolate for an additional 5 genes commonly used for choanoflagellate phylogeny reconstruction in [30]. We were able to retrieve the sequences of two genes, EFL and HSP90 via BLAST using the *S. rosetta* and *M. brevicollis* genes as queries and taking the top hit (which was the same for both query species in both cases). We incorporated these two genes into a concatenated three-gene phylogeny (Fig. 1C).

Prior to tree reconstruction, we processed each gene independently, as follows. For protein-coding genes (EFL and HSP90), we trimmed poly-A tails for all sequences using the program trimest from the EMBOSS package version 6.6.0.0 [31], with the '-nofiveprime' option and all other parameters left at their defaults. We aligned each gene separately using FSA version 1.15.7 [28] with the '--fast' option, and trimmed the resulting alignments using trimAl version 1.2rev59 [32] with the '-gt 0.3' option.

We concatenated trimmed sequences into a single alignment with three total partitions for tree reconstruction (following [30], a thorough exploration of choanoflagellate phylogenetics, which included the genes and choanoflagellate species we analyzed here, with the exception of *B. monosierra* and the three *S. rosetta* relatives that we added as described above): one partition for the 18S gene, one partition for the first and second codon positions in the protein-coding genes, and one partition for the third codon position. We reconstructed maximum likelihood phylogenies using RAxML version 8.2.4 [33], with the GTRCAT model and the '-f a -N 100' options for bootstrapping. We reconstructed Bayesian phylogenies with MrBayes version 3.2.6 [34], using a GTR + I + Γ model run for 1 million generations, with all other parameter values left at their defaults. The models chosen were according to the precedent set in reference [30]. For the phylogeny with different isolate 18S sequences, the final average standard deviation of split frequencies was 0.004327, and for the three-gene phylogeny for ML 2.1, it was 0.000074.

Phylogenetic analysis of bacteria

For generating the bacterial 16 ribosomal protein tree (Fig. 2E), a previously developed data set [35] was used. For each Mono Lake bacterial genome, 16 ribosomal proteins (L2, L3, L4, L5, L6, L14, L15, L16, L18, L22, L24, S3, S8, S10, S17, and S19) were identified by BLASTing [36] a reference set of 16 ribosomal proteins against the protein sets. (Dereplicated bin set used for constructing the ribosomal protein tree is summarized in Table S8.) BLAST hits were filtered to a minimum e-value of 1.0×10^{-5} and minimum target coverage of 25%. The resulting 16 ribosomal protein sets from each Mono Lake bacterial genome and the full reference set were aligned with MUSCLE (v. 3.8.31)[37]. Alignments were trimmed by removing columns containing 90% or greater gaps and then concatenated. A maximum likelihood tree was constructed using RAxML (v. 8.2.10)[33] on the CIPRES web server [38] with the LG plus gamma model of evolution (PROTGAMMALG) and the number of bootstraps automatically determined with the MRE-based bootstopping criterion.

For the bacterial 16S rRNA tree (Fig. S10), Mono Lake 16S rRNAs were aligned against the SILVA 128 SSU Ref NR 99 database [39] with BLAST [36] and the top three hits for each individual sequence, as well as a set of archaeal sequences for use as an outgroup, were aligned with MUSCLE (v. 3.8.31)[37][35][34][32]. For both the bacterial 16S rRNA trees, RAxML was used to construct a maximum likelihood tree with the GTRCAT model and the MRE-based bootstopping criterion.

Fluorescence *In Situ* Hybridization (FISH) Probe Design

Starting with the metagenome sequences described above, we developed FISH probes directed against the 16S sequence for each of the potential species detected in each sample. A detailed protocol for probe design using the ARB project software (http://www.arb-home.de/)[40] can be found at protocols.io at the following link: https://www.protocols.io/view/16s-rrna-probe-design-for-hcr-fish-wdffa3n. In short,16S rRNA sequences assembled from metagenomic sequencing utilizing EMIRGE above were imported and aligned to the SILVA 128 SSU Ref NR 99 database [39] in the ARB project using the automatic alignment tool. Probes were designed against individual bacteria identified in the *B. monosierra* samples containing choanoflagellate colonies (Fig. S11, Table S4) using the probe design tool and checked with the probe match tool in the ARB project software. The following HCR-amplification sequences were added to the 3' end of the probe sequences based on the fluorophore (488, 594, 647) and hairpins (B1, B2, B3) intended for the experiment: B1(488)(5'-ATATA GCATTCTTTCTTGAGGAGGGCAGCAAACGGGAAGAG-3'), B2(594)(5'-AAAAA AGCTCAGTCCAT CCTCGTAAATCCTCATCAATCATC-3), and B3(647)(5'-TAAAA AAAGTCTAATCCGTCCCTGCCTCTATATCTCCACTC-3'). Full length sequences of the probe with spacer and initiator sequences can be found in Table S6.

We performed an *in silico* probe specificity test against all of the sequences in the SILVA database, using TestProbe 3.0 from ARB-SILVA (https://www.arb-silva.de/?id=650). For the 11 species found inside B. monosierra colonies, all except for three had probes with 0 or 1 exact match in SILVA (Supplementary Data 1 – tab 1; zero exact matches are expected when our sequences are not present in the SILVA database.) The remaining three probes had 13, 3 and 6 exact hits. Taxonomic assignments for exact matches are listed in the table.

We also tested whether the probes we designed to target each potential species might also have off-target matches to other 16S sequences assembled by EMIRGE (Supplementary Data 1– tab 2). Not all target species have perfect matches to their probes. This is because the assembled EMIRGE contig lacks the probe sequence, but it is still classified as that species according to the full 16S phylogeny. The majority of the probes have no predicted perfect off-target matches. The exceptions are largely within the *Oceanospirillales* sp., where there are predicted off-target matches to other *Oceanospirillales* sp., and *Cytophagia* sp. 3-4, which has off-target effects versus *Cytophagia* sp. 1 and *Cytophagia* sp. 2 (for which probes were not designed). Allowing up to one mismatch to off-target sequences, there are only two cases in which the target bacterial species and the off-target bacterial species are differentially assigned to inside versus outside colonies. The first is *Marinospirillum* sp. 1-2, not considered to be inside colonies, with an off-target effect of its OG_Mar1 probe against *Oceanospirillales* sp. 5-6, which is assigned to inside colonies. The second is *Oceanospirillales* sp. 5-6, inside colonies, with an off-target effect of KH_293_OceaA203_C155.1 against D_15, which we could not assign to a species (it is an outgroup to *Oceanospirillales* in the tree, all of which are found inside colonies).

Fluorescence *In Situ* Hybridization and imaging

For the FISH experiments (Fig. 2C-C'''; Fig. S11-S13) we used hybridized chain reaction (HCR) – FISH. Our detailed protocol for HCR–FISH for choanoflagellate cultures can be found at protocls.io at the following link: https://www.protocols.io/edit/hcr-fish-for-choanoflagellate-cultures-wddfa26/steps. In short, the hairpin solutions and amplifier sequences used in this study were obtained from Molecular Instruments (www.molecularinstruments.com). *B.*

monosierra choanoflagellate cultures with free-living bacteria were fixed overnight in 2% paraformaldehyde at 4˚C. Cultures were filtered and mounted similar to traditional catalyzed reporter deposition (CARD) FISH methods [41,42]. To capture choanoflagellate colonies and free-living bacteria, fixed culture was filtered onto a 0.2 µm pore size 25 mm filter (Millipore Sigma, Darmstadt, Germany; Cat. No. GTTP02500). To capture only choanoflagellate colonies and let free-living bacteria pass through, cultures were filtered onto a 5 µm pore size 25 mm filter (Millipore Sigma; Cat. No. TMTP02500). Air-dried filters were coated in 0.1% low melt agarose and cut into wedges for hybridization experiments. To permeabilize bacterial cells, filters were incubated in a CARD-FISH proteinase buffer (10 mg/mL lysozyme; 0.05 M EDTA; 0.1M Tris-HCl, pH 8.0)[42] at 37˚C for 30 min. Filters were washed twice in nuclease-free H_2O, and once in 98% EtOH and left to air-dry. Filters were pre-hybridized in 1 mL of hybridization buffer (100 µl 20X SSC; 100 mg Dextran sulfate (Millipore Sigma; Cat. No. D6001); 200 µl Formamide (20% final conc.)[43] for 30min. at 45˚C. Filters were transferred into 500 µl of hybridized buffer with 0.25 µl of 100 mM stock HCR-FISH probes and incubated overnight at 45˚C. All filters were labeled with the universal EUB338 probe [44] to label all bacteria. Gam42a was used to label gammaproteobacteria (Fig. 2C')[45]. Custom probes (Table S4, Table S6) were used to label bacteria from *B. monosierra* cultures. Filters were washed in pre-warmed wash buffer based on the formamide concentration of the hybridization buffer (for 20% formamide hybridization: per 50 mL; 0.5 mL 0.5M EDTA, pH8.0; 1.0 mL 1M Tris HCl, pH8.0; 2150 µl 5M NaCl; 25 µl 20% SDS (w/v))[46]. To wash away unbound probes, filters were incubated in wash buffer for 1 hour at 48˚C followed by three washes for five minutes in 5X SSCT (per 40 mL; 10 mL 20X SSC; 400 µl 10% Tween 20). Filters are incubated in amplification buffer (for 40 mL; 10 mL 20X SSC; 8 mL 50% Dextran Sulfate; 400 µl 10% Tween 20)[47] for 30 minutes at room temperature while hairpin solutions were snap cooled as previously described [47]. Signal amplification was performed by incubating filters in amplification buffer with hairpins overnight in the dark in a humidified chamber. To wash, filters were placed in amplification buffer for 1 hour in the dark at room temperature followed by two washes for 30 minutes in 5X SSCT in the dark at room temperature. To stain DNA, filters were washed for 10 minutes in 5X SSCT with 0.1mg/mL Hoechst 33342 (Thermo Fisher Scientific). Finally, filters were washed for 1 minute in nuclease free H_2O, and 1 minute in 96% EtOH before air drying and mounting in ProLong Diamond (Thermo Fisher Scientific). Slides were left overnight to cure before imaging on a Zeiss Axio Observer LSM 880 with Airyscan detector as described above in confocal imaging. The bacteria SaccML, OceaML1, OceaML2, EctoML1, EctoML2, EctoML3, and RoseML were identified in the culture ML2.1EC. OceaML3, OceaML4, and EctoML4 were identified in the culture ML2.1E.

Quantitative image analysis of FISH data set

FISH was performed as described above to label specific bacteria (SaccML, OceaML1, OceaML2, EctoML1, EctoML2, EctoML3, and RoseML) along with a broad-spectrum bacterial probe EUB 338 [44]. All experiments for quantitative image analysis (Fig. S14) were performed in the culture ML2.1EC. A minimum of 120 rosettes were imaged at random per phylotype on 5 µm filters using a 63X/NA 1.15 oil immersion objective on a Zeiss LSM 880 AxioExaminer. A z-stack was acquired for each rosette to capture the whole rosette. Images were analyzed on FIJI [13]. Due to the pressure applied to the rosettes during the filtering process, colonies are flattened; therefore, we applied a maximum projection for each z-stack leaving out slices that contained the filter. Colonies were first assessed to see if the phylotype was present. If at least one bacterial phylotype was found inside the colony, the colony was counted as having the phylotype. Due to the HCR-FISH method, single bacterial resolution is possible (Fig. S12). To determine the abundance of each phylotype present in a colony, colony images were cropped down to only contain the interior bacteria, and then the images were split into separate

channels: EUB 338 bacterial probe, phylotype-specific probe, and Hoechst 33342. The Hoechst image was thrown out because it was not needed. An automatic threshold using the Intermodes method [14] was applied to both the broad-spectrum bacterial probe and bacterial phylotype-specific probe images. The area was measured for each threshold (total bacteria broad spectrum probe and phylotype-specific probe) by setting measurements to area and analyzing the images. The area occupied by a particular phylotype was divided by the area of the total bacteria for each colony to determine the proportion or abundance of each bacterial phylotype present in individual colonies.

Gammaproteobacteria and Alphaproteobacteria live in *B. monosierra* colonies

We sought to identify which bacterial phylotypes comprise the bacterial communities of lab-reared *B. monosierra.* To identify candidate bacteria for which to design FISH probes, we sequenced and assembled metagenomes and 16S rRNA sequences from choanoflagellate-enriched and environmental bacteria-enriched samples. These samples were derived from two co-cultures of *B. monosierra* with Mono Lake bacteria, ML2.1E and ML2.1G (Fig. S1), with the enrichment for choanoflagellates or bacteria performed by centrifugation. A total of 24 different bacterial phylotypes were identified using two complementary bioinformatic approaches (Genome-resolved metagenomic analysis and EMIRGE 16S rRNA Analysis; Supplemental Methods; Table S2 and S3), of which 22 phylotypes were present in fractions enriched with *B. monosierra* rosettes (Table S4).

The 22 bacterial phylotypes detected in cultures with *B. monosierra* may have co-sedimented with the *B. monosierra* rosettes due to their community-structure densities (e.g., biofilms), a transient association with the choanoflagellate rosettes (e.g., as prey), or through a stable association with the choanoflagellate rosettes. We therefore designed FISH probes to investigate the spatial distribution of candidate *B.* monosierra-associated bacterial phylotypes (see Text S1 for a description of probe design and tests of probe specificity).

Upon investigation by FISH microscopy, we detected ten or eleven of these phylotypes in the centers of *B. monosierra* rosettes (Table S4, Fig. S11). (The uncertainty regarding the precise number of choanoflagellate-associated bacterial species stems from the inability to disambiguate 16S rRNA sequences corresponding to one or two species.) Of these bacteria, nine were Gammaproteobacteria from the families *Oceanospirillaceae* (Fig. S11A; OceaML1, OceaML2, OceaML3, OceaML4, OceaML4)*, Ectothiorhodospiraceae* (Fig. S11B; EctoML1, EctoML2, EctoML3, EctoML4), and *Saccharospirillaceae* (Fig. S11C; SaccML), matching our original observation that the majority of the bacteria were Gammaproteobacteria (Fig. 2C, C’). The remaining phylotype was a *Roseinatronobacter* sp. (RoseML; Alphaproteobacteria) (Fig. S11D).

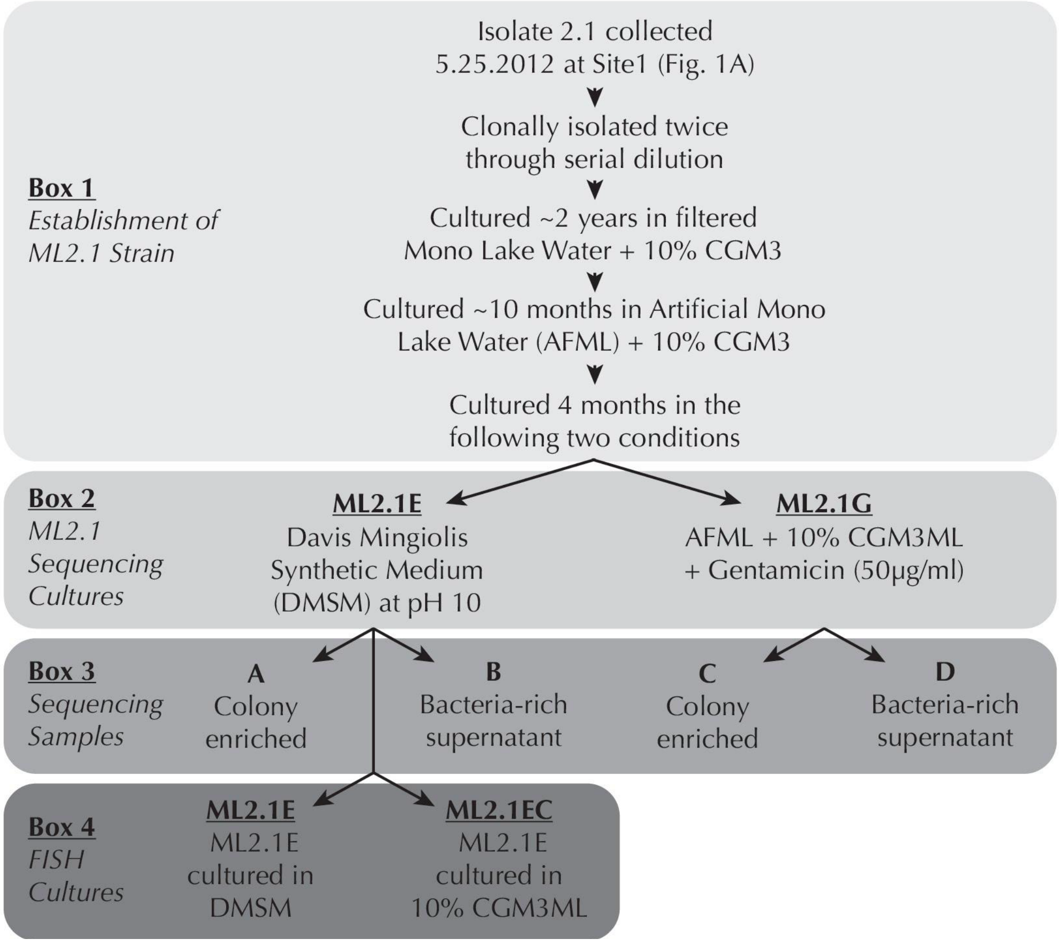

Figure S1: Establishment of *B. monosierra* cultures. ML2.1E and ML2.1G were derived from the original isolate ML2.1 used in the sequencing experiment to identify bacteria present in the *B. monosierra* colonies, as well as, outside of the colonies. (Box 1) Isolate 2.1 was collected on 5.25.2012 at Site 1 (Fig. 1A, Table S1). Each isolate was clonally isolated by serial dilution twice, and ML2.1 was cultured in the lab for nearly 2 years in filtered Mono Lake water supplemented with cereal grass media (CGM3) concentrate as a carbon source. Upon generating an artificial Mono Lake water (AFML), ML2.1 was cultured for 10 months in AFML with CGM3ML. The ML2.1 culture was then split into two cultures (ML2.1E and ML2.1G, Box 2). ML2.1E was cultured in Davis Mingiolis Synthetic minimal medium at pH10 and ML2.1G was similar to ML2.1 cultured in AFML with the addition of a six-week Gentamicin antibiotic treatment that did not affect colony size and improved overall growth of the culture. (Box 3) Through differential centrifugation and filtration, a sample enriched for *B. monosierra* colonies and the supernatant enriched with bacteria were used for genomic DNA extraction for each culture, ML2.1E and ML2.1G. (Box 4) Due to variability in ML2.1E rosette development, likely due to the diversity and complicated growth dynamics of prey bacteria, we found maintaining a culture under multiple growth conditions increased our chances of having a culture with large *B. monosierra* rosettes. The ML 2.1E culture was grown further in DMSM (ML 2.1E) and in 10% CGM3ML (ML 2.1EC); the resulting cultures were used for FISH analysis.

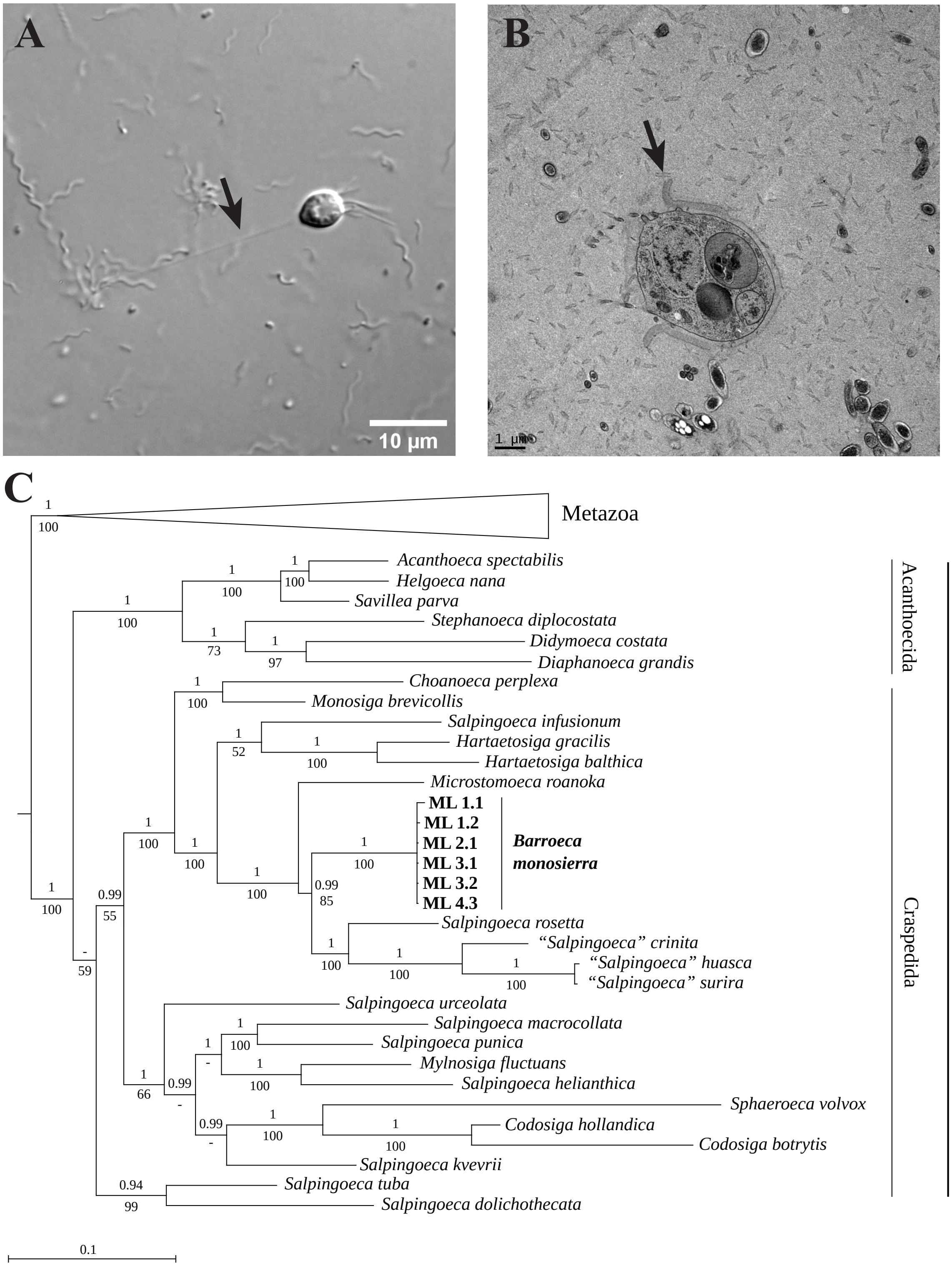

Figure S2: Ultrastructure and phylogeny of choanoflagellate isolates from Mono Lake.
A. Single cells may be found attached to a substrate via a long (~30 µm) basal pedicel (arrow). **B**. Single cells may possess an organic cup-shaped theca with a distinctive ~0.75 µm outward-facing lip on its apical end (arrow). **C**. Phylogenetic tree of sequences from 6 Mono Lake choanoflagellate isolates (labeled on tree) and other choanoflagellate species reveal the isolates (Table S1) are

members of the same species. The tree includes data from 3 genes (18S, EFL and HSP90) as a backbone, with each Mono Lake isolate represented only by its 18S sequence. ML 2.1 is the primary strain used in this publication. Metazoa (7 species) were collapsed to save space. Bayesian posterior probabilities are indicated above each internal branch, and maximum likelihood bootstrap values below. (A ‘-’ value indicates a bifurcation lacking support or not present in one of the two reconstructions).

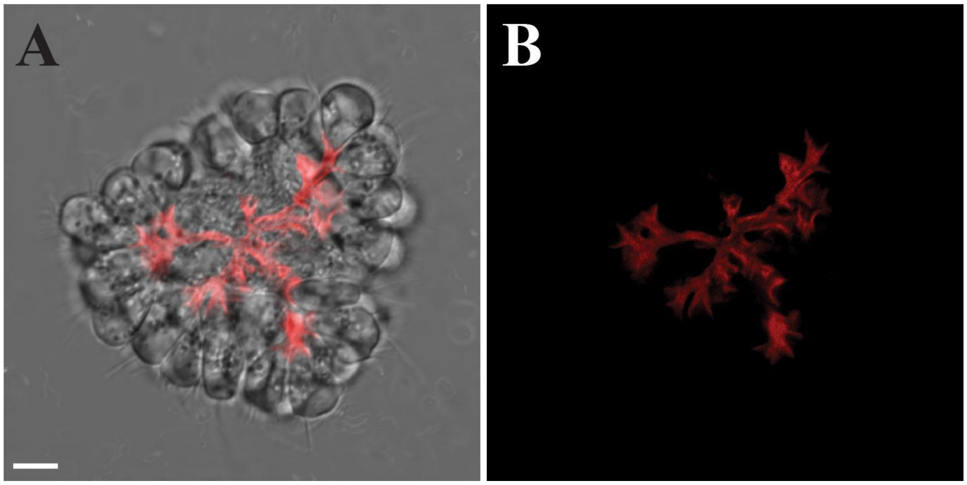

Figure S3. *B. monosierra* colonies contain an extracellular matrix (ECM).
(A, B) *B. monosierra* colonies contain a branched network of ECM that extends from and connects the basal pole of cells in the rosette. Optical section of representative colony (from culture ML2.1G) (A), stained with the lectin Concanavalin A (B; red), shown.

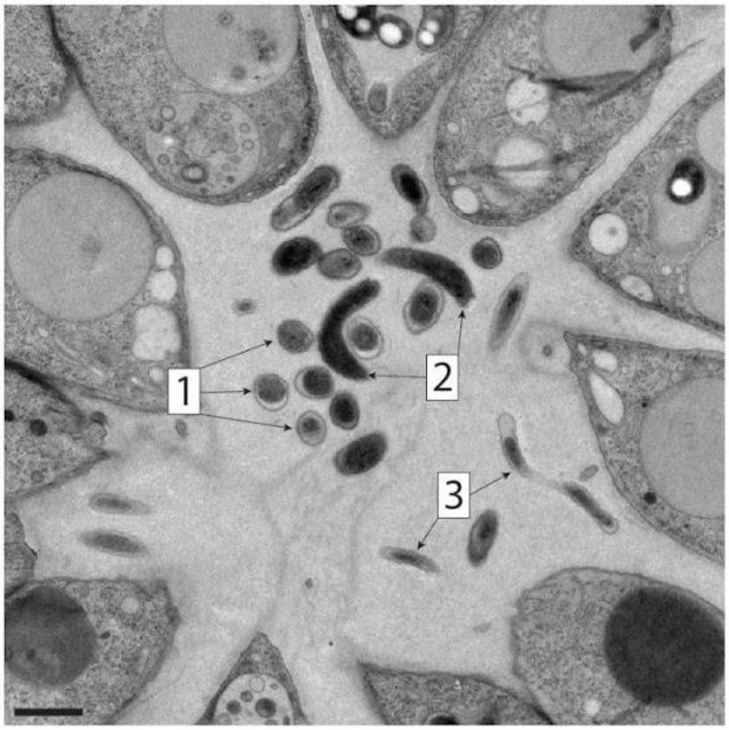

Figure S4: Bacterial residents in *B. monosierra* rosettes exhibit a range of morphologies. Bacteria inside the rosettes of *B. monosierra* have at least 3 distinct morphologies (1-3) revealed by TEM. Scale bar = 1 μm.

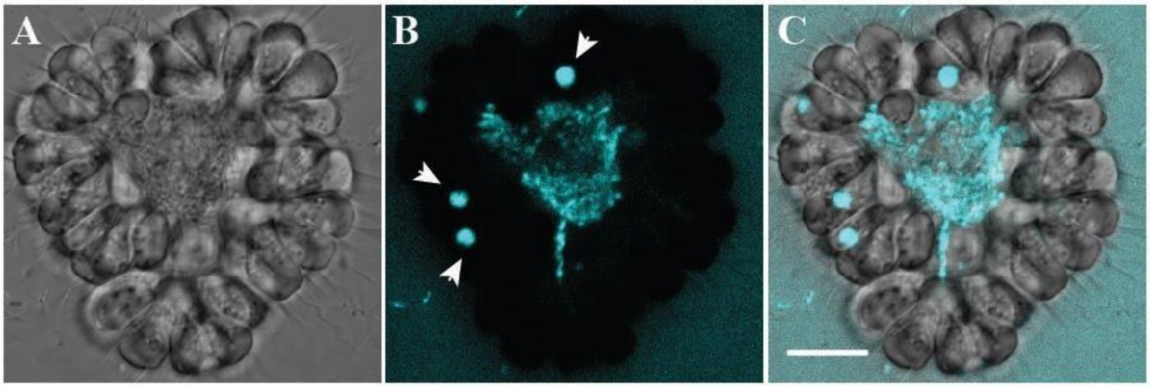

Figure S5: Bacteria inside *B. monosierra* rosettes are alive and growing.
(A) Optical section of colony imaged by DIC. (B) Bacterial cells inside *B. monosierra* colonies incorporate fluorescent D-amino acids into their cell walls (cyan)[15], indicating that the bacteria are alive and actively growing. The D-amino acids also accumulate in the food vacuoles of the choanoflagellate cells (arrowheads) through phagocytosis of the dye and of labeled bacteria from outside the colony. Overlay (C) shows D-amino acid enrichment inside the colony, corresponding to the location and morphology of the bacteria in the lumen. Scale bar = 10 μm.

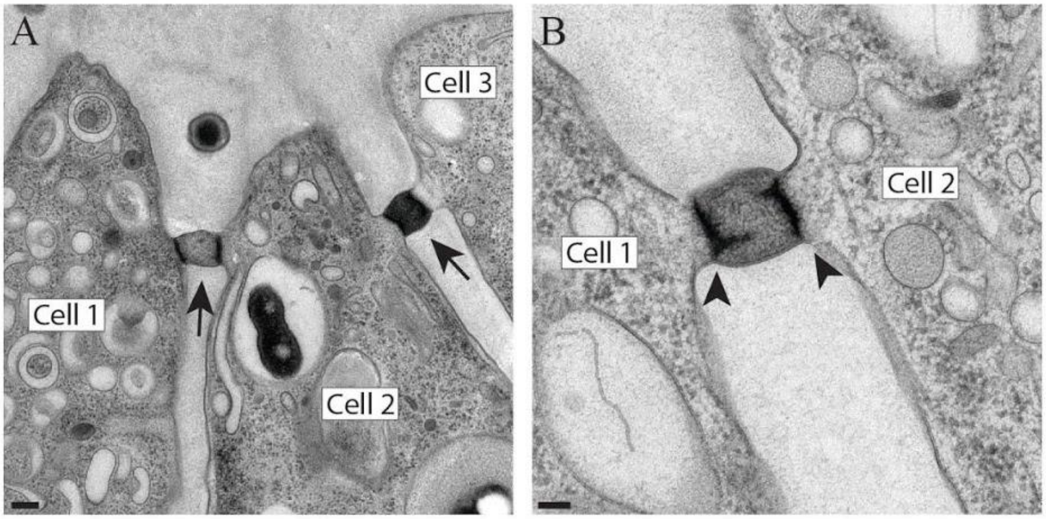

Figure S6: Intercellular bridges connect cells in *B. monosierra* rosettes.
(A) Many cells in *B. monosierra* colonies are connected by intercellular bridges (arrows) that resemble the bridges found in *S. rosetta* colonies [16] and other choanoflagellates [48,49]. Choanoflagellate cells labeled with boxes. (Cell 2 contains a phagocytosed bacterium in its food vacuole. This is not to be confused with the extracellular/luminal bacterial communities documented in Figs. 1, 2, and S3.) Scale bar = 200nm. (B) TEM of intercellular bridges between two choanoflagellate cells reveals two electron dense plates (arrowheads) in an arrangement that is reminiscent of the ultrastructure of *S. rosetta* bridges [16]. Scale bar = 100nm.

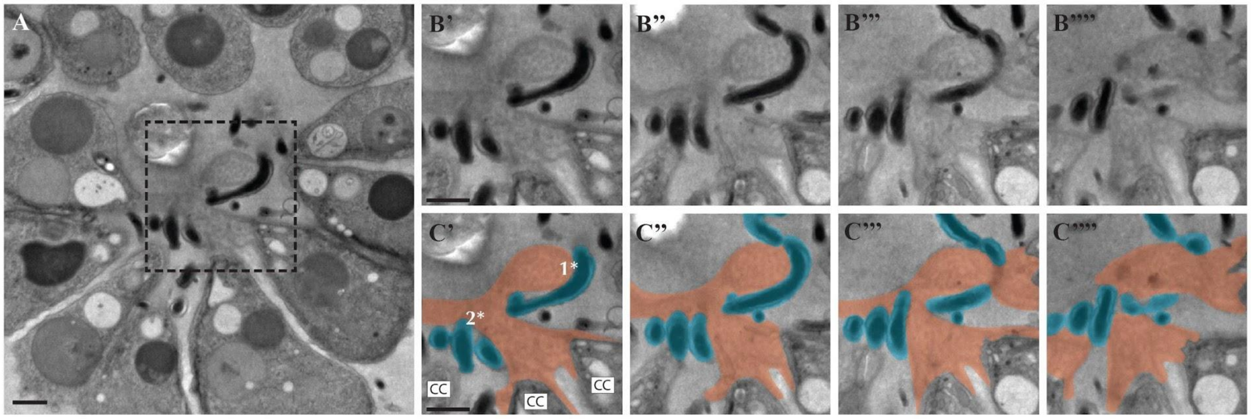

Figure S7: Bacterial residents physically associate with and wrap around the choanoflagellate ECM.
(A) A single TEM section through a *B. monosierra* rosette shows choanoflagellate cells on the periphery and bacteria in the center. Box indicates region shown in panels B – C. (B, C) Serial sections (150 nm) through the colony reveal the close proximity of bacteria to the choanoflagellate ECM. (C) False coloring of the TEM sections highlights the associations among the ECM (orange) and bacteria (blue) that wrap around the ECM at two separate sites (1*, 2*). Choanoflagellate cells indicated as CC. Scale bar = 1 µm.

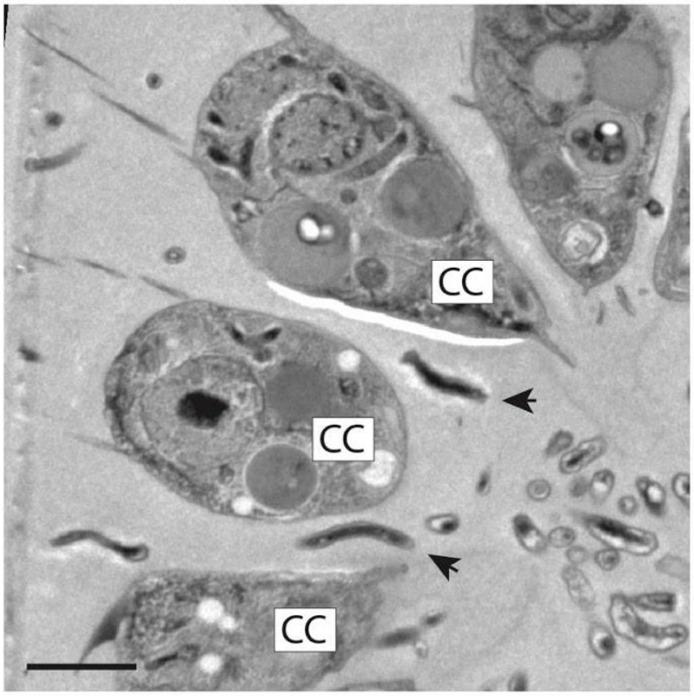

Figure S8: Bacteria are found wedged between the lateral surfaces of *B. monosierra* cells. Bacteria (arrowheads) were observed between cells of *B. monosierra* rosettes. TEM image illustrates two spiral bacteria (arrowheads) positioned between choanoflagellate cells (CC). Scale bar = 2 μm.

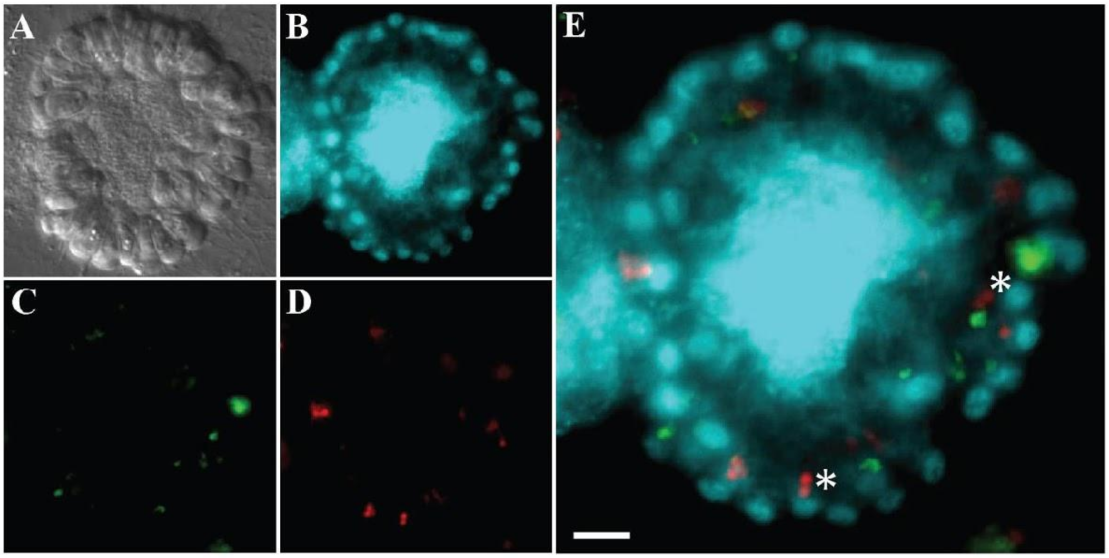

Figure S9: ***B. monosierra*** **rosettes cannot be passively penetrated by sub-micron particles.** (A-E) *B. monosierra* colonies fail to incorporate bacteria-sized microspheres over a 24-hour incubation time, suggesting that bacteria cannot enter the colony center passively. An optical section through a *B. monosierra* colony (A, DIC; B, Hoechst), illuminates the spherical choanoflagellate nuclei and interior bacteria. The fluorescent beads (0.22 μm, green, C; 1 μm, red, D) were never observed in the interior cavities of the colonies. They were observed in food vacuoles of choanoflagellate cells (E, asterisks) due to phagocytosis of the beads through the same pathway used for phagocytosis of bacterial prey. Scale bar = 5 μm.

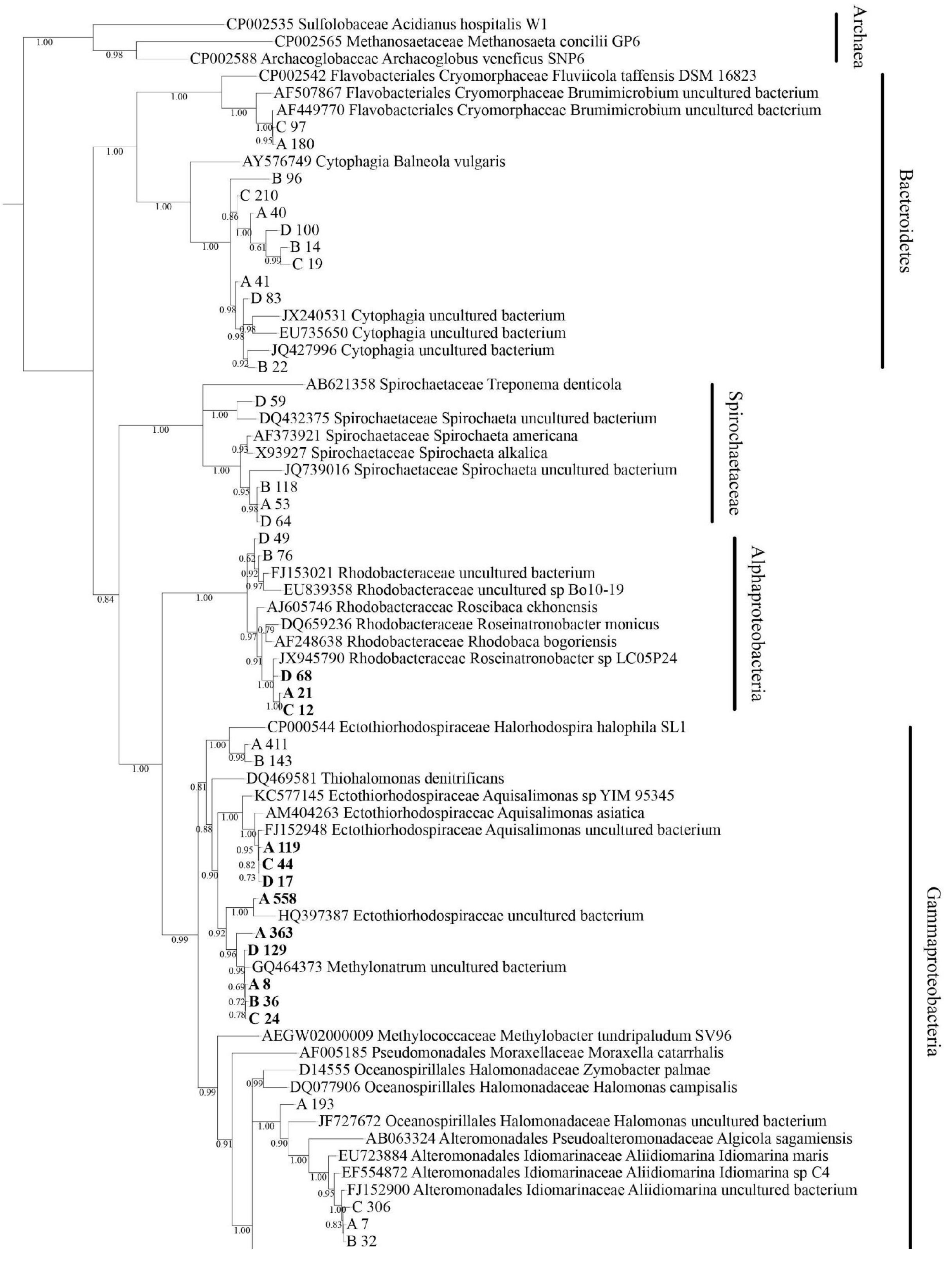
CP002535 Sulfolobaceae Acidianus hospitalis W1
CP002565 Methanosaetaceae Methanosaeta concilii GP6
CP002588 Archaeoglobaceae Archaeoglobus veneficus SNP6
CP002542 Flavobacteriales Cryomorphaceae Fluviicola taffensis DSM 16823
AF507867 Flavobacteriales Cryomorphaceae Brumimicrobium uncultured bacterium
AF449770 Flavobacteriales Cryomorphaceae Brumimicrobium uncultured bacterium
C 97
A 180
AY576749 Cytophagia Balneola vulgaris
B 96
C 210
A 40
D 100
B 14
C 19
A 41
D 83
JX240531 Cytophagia uncultured bacterium
EU735650 Cytophagia uncultured bacterium
JQ427996 Cytophagia uncultured bacterium
B 22
AB621358 Spirochaetaceae Treponema denticola
D 59
DQ432375 Spirochaetaceae Spirochaeta uncultured bacterium
AF373921 Spirochaetaceae Spirochaeta americana
X93927 Spirochaetaceae Spirochaeta alkalica
JQ739016 Spirochaetaceae Spirochaeta uncultured bacterium
B 118
A 53
D 64
D 49
B 76
FJ153021 Rhodobacteraceae uncultured bacterium
EU839358 Rhodobacteraceae uncultured sp Bo10-19
AJ605746 Rhodobacteraceae Roseibaca ekhonensis
DQ659236 Rhodobacteraceae Roseinatronobacter monicus
AF248638 Rhodobacteraceae Rhodobaca bogoriensis
JX945790 Rhodobacteraceae Roseinatronobacter sp LC05P24
D 68
A 21
C 12
CP000544 Ectothiorhodospiraceae Halorhodospira halophila SL1
A 411
B 143
DQ469581 Thiohalomonas denitrificans
KC577145 Ectothiorhodospiraceae Aquisalimonas sp YIM 95345
AM404263 Ectothiorhodospiraceae Aquisalimonas asiatica
FJ152948 Ectothiorhodospiraceae Aquisalimonas uncultured bacterium
A 119
C 44
D 17
A 558
HQ397387 Ectothiorhodospiraceae uncultured bacterium
A 363
D 129
GQ464373 Methylonatrum uncultured bacterium
A 8
B 36
C 24
AEGW02000009 Methylococcaceae Methylobacter tundripaludum SV96
AF005185 Pseudomonadales Moraxellaceae Moraxella catarrhalis
D14555 Oceanospirillales Halomonadaceae Zymobacter palmae
DQ077906 Oceanospirillales Halomonadaceae Halomonas campisalis
A 193
JF727672 Oceanospirillales Halomonadaceae Halomonas uncultured bacterium
AB063324 Alteromonadales Pseudoalteromonadaceae Algicola sagamiensis
EU723884 Alteromonadales Idiomarinaceae Aliidiomarina Idiomarina maris
EF554872 Alteromonadales Idiomarinaceae Aliidiomarina Idiomarina sp C4
FJ152900 Alteromonadales Idiomarinaceae Aliidiomarina uncultured bacterium
C 306
A 7
B 32
Archaea
Bacteroidetes
Spirochaetaceae
Alphaproteobacteria
Gammaproteobacteria

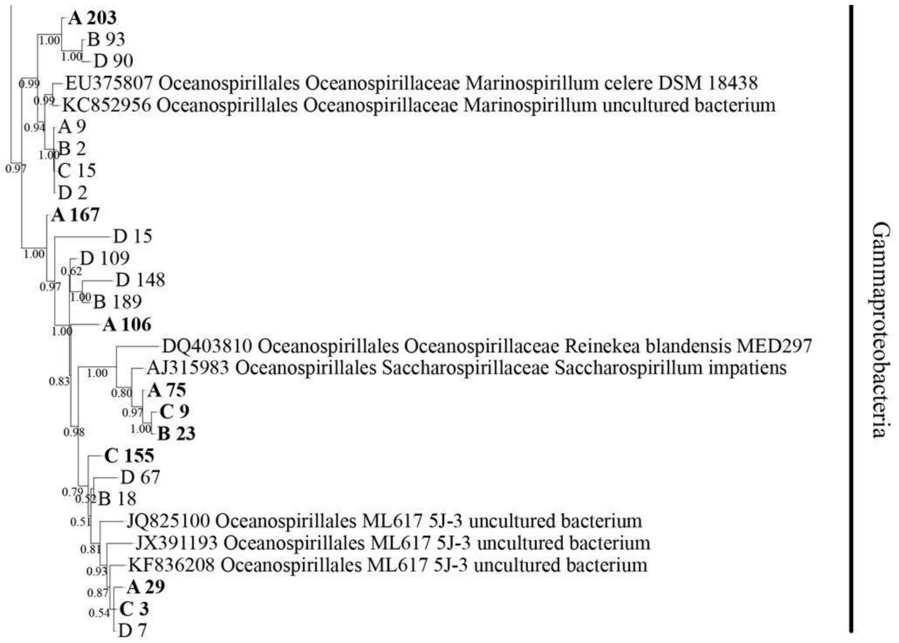

Figure S10: Phylogenetic analysis of 16S rRNA sequences.
The tree was built by comparing 16S rRNA sequences assembled from the Mono Lake samples through EMIRGE analysis with 16S rRNA sequences from their closest relatives. Sequences from Mono Lake samples are labeled by the letter associated with the sequencing sample (A or C: with colonies; B or D: environmental bacteria; Fig. S9, Box 3) and a unique identifying number. Species shown in bold were detected inside rosettes (Table S4). Reference species are shown with an accession number and genus name, along with other identifying information. Bootstrap values displayed. Branches with <0.5 bootstrap value have been collapsed.

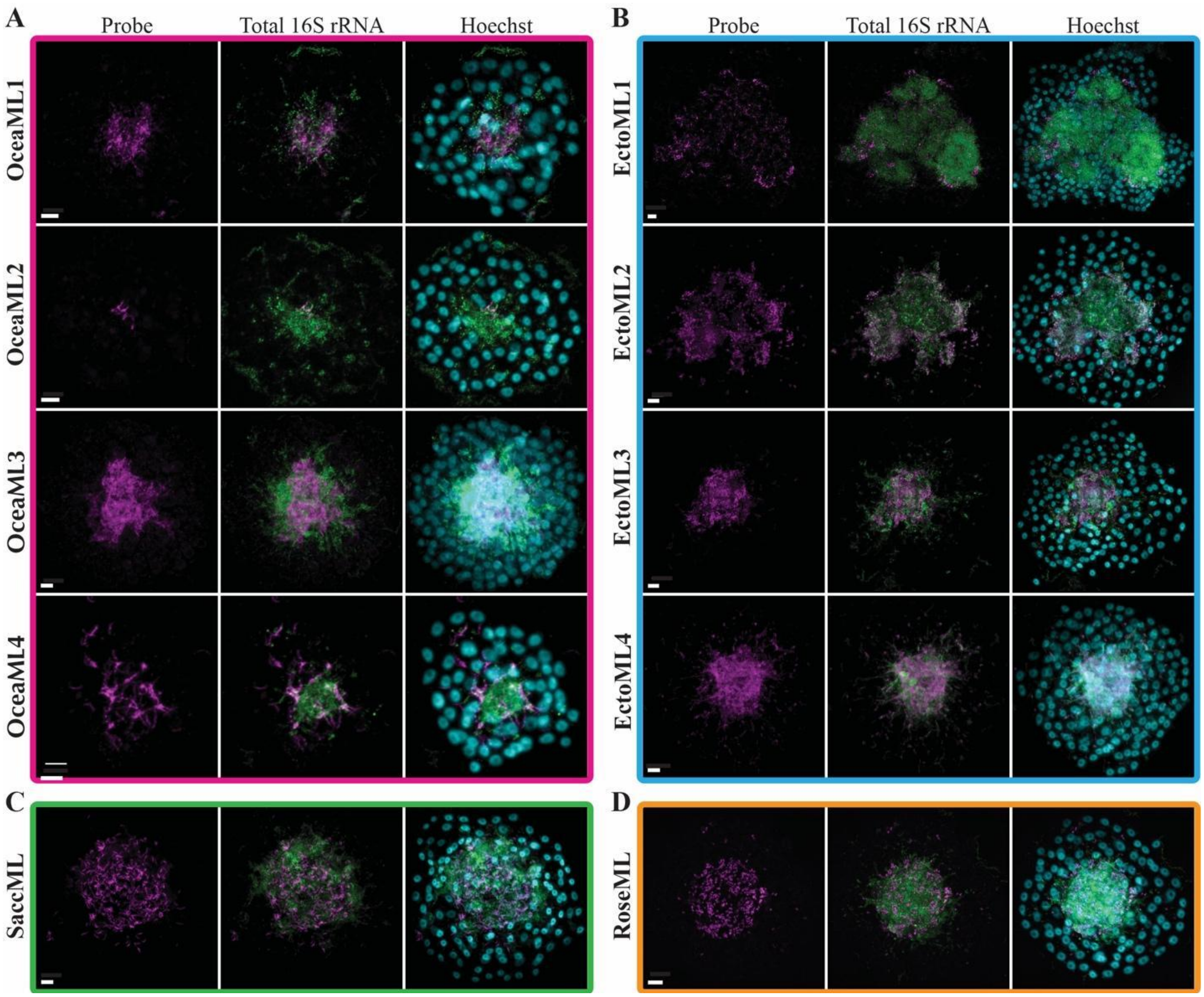

Figure S11: Diverse bacteria are found in the lumen of *B. monosierra.*
(A-D) Ten different bacterial species detected in the center of *B. monosierra* colonies. Confocal images of representative colonies hybridized with phylotype-specific probes (magenta; Table S4, Table S6) and a broad spectrum 16S rRNA probe (green) overlaid with Hoechst 33342 staining of the choanoflagellate nuclei and bacterial nucleoids (cyan). The bacteria are grouped by genus and color coded to match the phylogenetic tree in Fig. 2E. The bacteria have been named as follows: (A) OceaML1 = *Oceanospirillaceae* sp.1; OceaML2 = *Oceanospirillaceae* sp. 2; OceaML3 = *Oceanospirillaceae* sp.3; OceaML4 = *Oceanospirillaceae* sp. 4; (B) SaccML = *Saccharospirillaceae* sp.; (C) EctoML1 = *Ectothiorhodospiraceae* sp. 1; EctoML2 = *Ectothiorhodospiraceae* sp. 2; EctoML3 = *Ectothiorhodospiraceae* sp. 3; EctoML4 = *Ectothiorhodospiraceae* sp. 4. and (D) RoseML = *Roseinatronobacter* sp. The bacteria SaccML, OceaML1, OceaML2, EctoML1, EctoML2, EctoML3, and RoseML were identified in the culture ML2.1EC. OceaML3, OceaML4, and EctoML4 were identified in the culture ML2.1E. Scale bars = 5 μm.

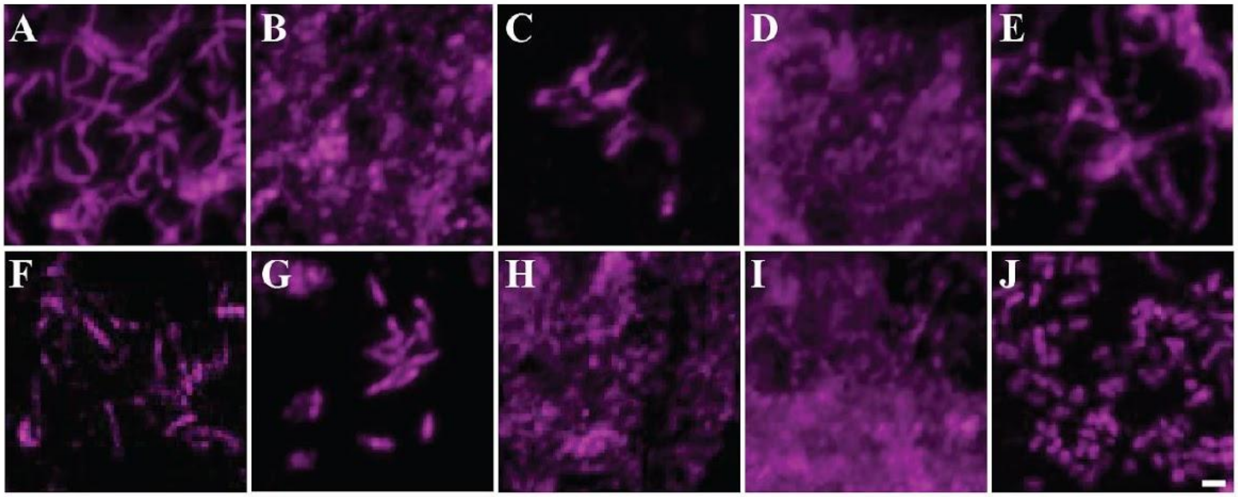

Figure S12: Resident bacteria of *B. monosierra* exhibit filamentous and rod morphologies. HCR-FISH probes illuminate distinct bacterial morphologies, from filamentous bacteria (A, C, E, F) to rod-shaped bacteria (B, G, J). (A) SaccML, (B) OceaML1, (C) OceaML2, (D) OceaML3, (E) OceaML4, (F) EctoML1, (G) EctoML2, (H) EctoML3, (I) EctoML4, (J) RoseML. Scale bar = 1 μm.

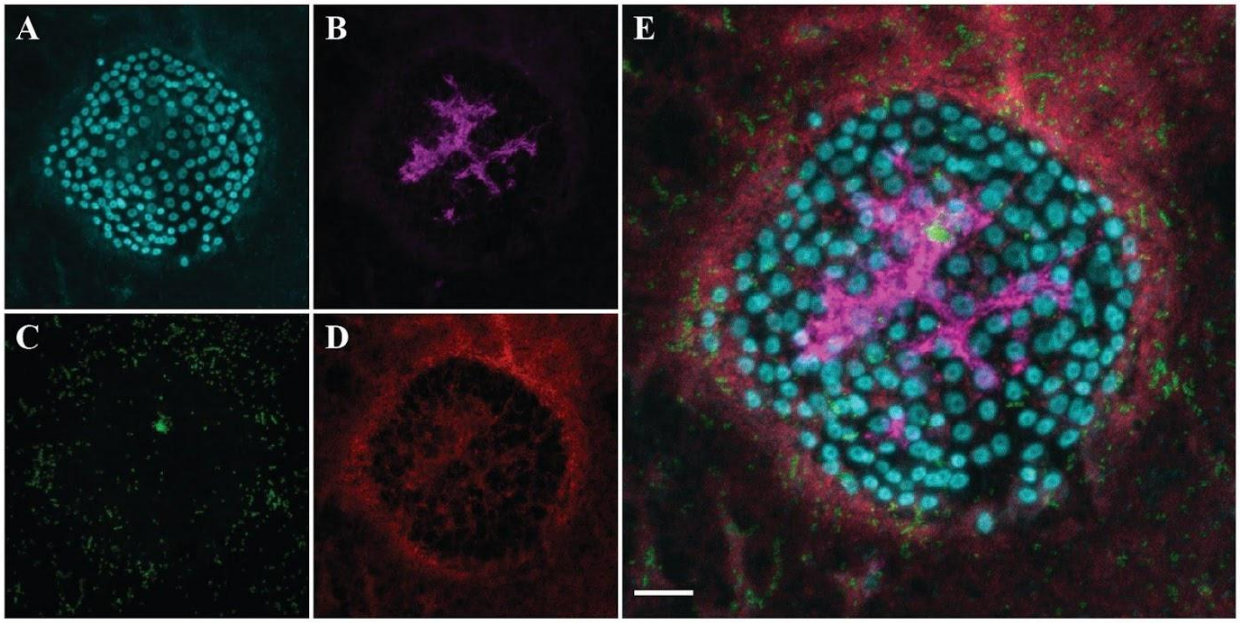

Figure S13: The bacterium OceaML3 was detected exclusively inside rosettes.
The *B. monosierra* colony identified by the cluster of spherical nuclei (A, cyan), shows how OceaML3 (B, magenta) is present exclusively inside the rosette whereas EctoML2 (C, green) and EctoML4 (D, red) can be detected both inside and outside the rosette. (E) Merge of all four channels. FISH analysis was performed on a 0.22 μm filter to capture free-living bacteria. Scale bar = 10 μm.

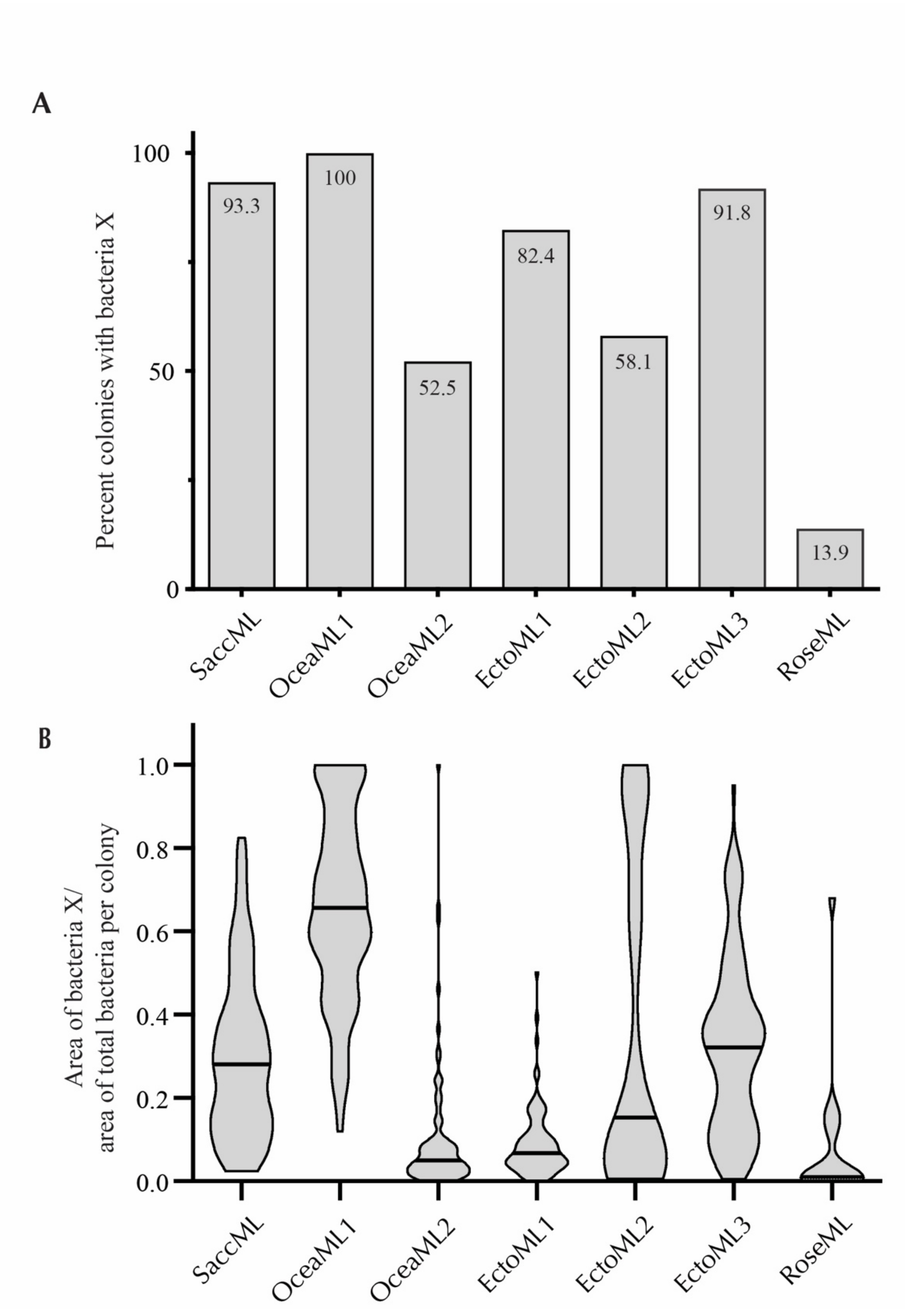

Figure S14: OceaML1 is a core member of the *B. monosierra* bacterial community. Frequency of bacteria (A) and their relative abundance inside the rosette (B) illustrates that each bacterium has a unique pattern. (A) The presence of each phylotype was assessed for a minimum of 120 rosettes. HCR-FISH can resolve individual bacteria (Fig. S12), and for the purposes of this analysis, a single positive cell was considered evidence of phylotype presence. (B) The area of the bacteria compared to the total area was used to determine the relative abundance. The data were graphed as a violin plot with the mean indicated as the black line. All choanoflagellates analyzed were from culture ML2.1EC (see Fig. S1, box 4).

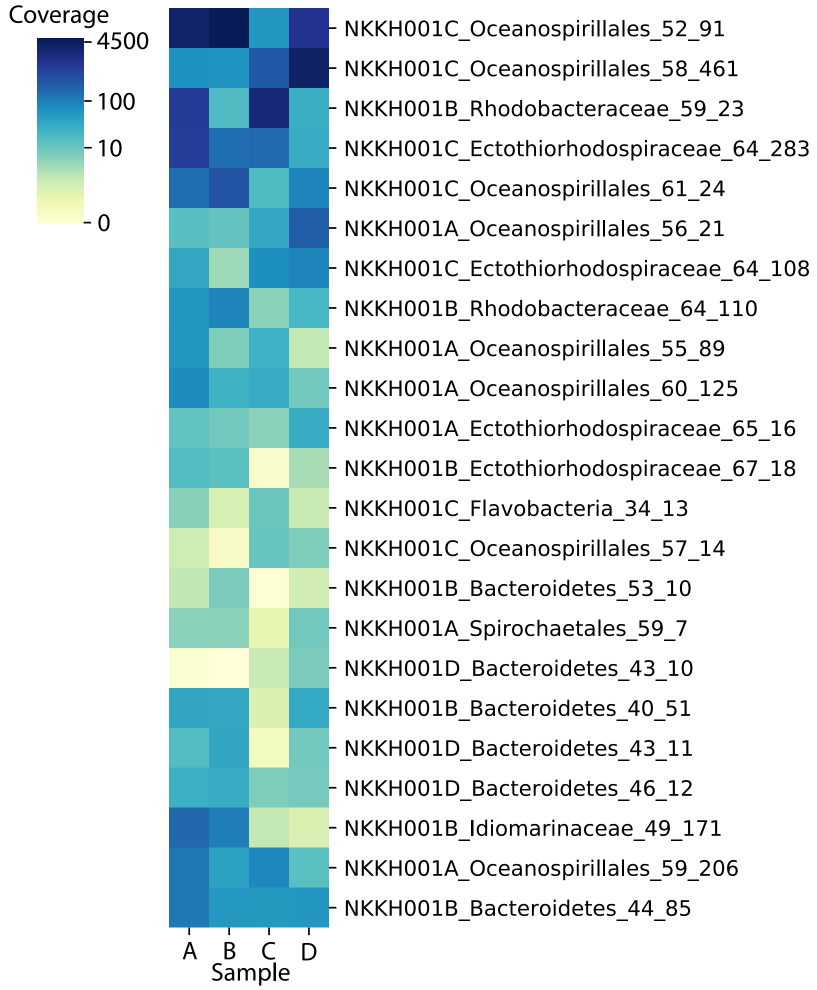

Figure S15: Bacterial community overlap across shotgun metagenomic sequencing samples. Sequencing reads from each shotgun metagenome were cross-mapped against the dereplicated bin set. Coverage for each bin was determined by averaging the coverage of a given bin’s constituent contigs.

Table S1: Date, location, phenotype, and isolate designations for *Barroeca monosierra* isolates.

Isolate	Date collected	Location**	18S Sequenced	% Identity with ML2.1	Phenotype when isolated	Phenotype after culturing in lab
ML 1.1	05.15.2012	Site 1	Yes	99.6%	Single Celled	Colonies
ML 1.2	05.15.2012	Site 1	Yes	99.4%	Single Celled	Colonies
ML 2.1*	05.25.2012	Site 1	Yes	100%	Colonies	Colonies
ML 3.1	05.05.2013	Site 1	Yes	99.3%	Colonies	Colonies
ML 3.2	05.05.2013	Site 1	Yes	99.3%	Colonies	Colonies
ML 3.3	05.05.2013	Site 1	No		Colonies	Colonies
ML 4.1	10.11.2014	Site 2	No		Colonies	Colonies
ML 4.2	10.11.2014	Site 2	No		Colonies	Colonies
ML 4.3	10.11.2014	Site 2	Yes	99.5%	Colonies	Colonies
ML 4.4	10.11.2014	Site 2	No		Colonies	Colonies
Wild colonies	06.17.2024	Site 3	No		Colonies	N/A

* Primary strain used in this publication

** See Fig. 1A. Site 1 is at the Mono Lake picnic area approximately at 37˚58’42.7”N 119˚01’52.9”W. Site 2 is at the Mono Lake South Tufa area approximately at 37˚56’37.1”N 119˚01’37.8”W. Site 3 is near the Mono Lake Black Point Parking Lot approximately at 38°01'26.9"N 119°04'49.8"W.

Table S2: Shotgun metagenomic sequencing project outcomes

	Sample			
	**A**	**B**	**C**	**D**
Sequencing Depth (Gbp)	34.1	25.8	31.3	22.6
Assembly size (Mbp)	173.7	86.7	141.6	87.4
EukRep removed sequence (Mbp)	50.3	NA	51.7	NA
# bins	17	12	9	10
# bins (dereplicated)	6	7	7	3

Table S3: Nine genera of bacteria identified in *B. monosierra* cultures through two independent analyses: comparison of ribosomal proteins detected through metagenomic assembly and by 16S rRNA assembly and analysis.

	**EukRep Metagenomic Analysis***		**EMIRGE 16S rRNA Analysis***	
**Class**	**Genus**	**Number of phylotypes**	**Genus**	**Number of phylotypes**
Spirochaetia	*Spirochaetia*	2	*Spirochaetia*	2
Gammaproteobacteria	*Oceanospirillaceae*	6	*Oceanospirillaceae*	6
	Saccharospirillaceae	1	*Saccharospirillaceae*	1
	Ectothiorhodospiraceae	4	*Ectothiorhodospiraceae*	5
	Idiomarinaceae	1	*Idiomarinaceae*	1
	Marinospirillum	1	*Marinospirillum*	2
Alphaproteobacteria	*Rhodobacteraceae*	2	*Rhodobacteraceae*	2
Bacteroidetes	*Chitonophagaceae*	6	*Chitonophagaceae*	4*
	Fluviicola	1	*Fluviicola*	1
	Total	24	Total	24

*See methods for detailed explanation of the relative challenges in using either solely 16S rRNA analysis or metagenomic analysis to detect uncharacterized bacteria from environmental samples.

Table S4: Predicted targets of HCR-FISH probes based on 16S rRNA sequences. Bacteria in bold were identified inside *B. monosierra* colonies.

Bacteria *	Probe name	Target 16S EMIRGE Sequence**
Saccharospirillum	KH 133_Sacc3	ML_16S_A_75, ML_16S_B_23, ML_16S_C_9
Idiomarinaceae	OG_Idio1	ML_16S_A_7, ML_16S_B_32, ML_16S_C_306
Marinospirillum 1	OG_Mar1	ML_16S_A_193
Marinospirillum 2	OG_Mar1	ML_16S_A_9, ML_16S_B_2, ML_16S_C_15, ML_16S_D_2
Oceanospirillales 1	KH 270_OceaA167.3	ML_16S_A_167
Oceanospirillales 2	KH 278_OceaA29.1	ML_16S_A_29
Oceanospirillales 3	KH 295_OceaC3.5	ML_16S_C_3
Oceanospirillales 4-5*	KH 288_ OceaA106_C155	ML_16S_A_106, ML_16S_C_155
Oceanospirillales 5-6*	KH 293_ OceaA203_C155.1	ML_16S_A_203, ML_16S_C_155
Ectothiorhodospiraceae 1	KH 168_Ecto8	ML_16S_A_363
Ectothiorhodospiraceae 2	KH 171_Ecto11	ML_16S_A_8, ML_16S_B_36, ML_16S_C_24, ML_16S_D_129
Ectothiorhodospiraceae 3	KH 174_Ecto14	ML_16S_A_119, ML_16S_C_44, ML_16S_D_17
Ectothiorhodospiraceae 4	KH 297_EctoA558.4	ML_16S_A_558
Ectothiorhodospiraceae 5	KH 285_EctoA411.3	ML_16S_A_411, ML_16S_B_143
Roseinatronobacter	KH 152_Rose3	ML_16S_A_21, ML_16S_C_12, ML_16S_D_68

Rhodobacterales	KH 154_Rhod2	ML_16S_B_76,<br>ML_16S_D_49
Cytophagia 1	***	ML_16S_B_96
Cytophagia 2	***	ML_16S_B_22,<br>ML_16S_D_83
Cytophagia 3-4 ****	KH 177_Cyto2	ML_16S_A_40,<br>ML_16S_A_41,<br>ML_16S_B_14,<br>ML_16S_C_19,<br>ML_16S_C_210,<br>ML_16S_D_100
Brumimicrobium	KH 124_Brum3	ML_16S_A_180,<br>ML_16S_C_97
Spirochaetia	KH 148_Spir2	ML_16S_A_53,<br>ML_16S_B_118,<br>ML_16S_D_64

** Target 16S rRNA sequences from EMIRGE analysis. A and C sequences are from *B. monosierra* colony-enriched samples while B and D sequences are from the bacteria-rich supernatant.
*** Bacteria identified only in bacteria-rich supernatant and not in colony-enriched sequences.
**** Exact number of phylotypes is undetermined due to sequence similarity in 16S rRNA EMIRGE assembly from 150bp Illumina sequencing reads.

Table S5: Bacterial 16S rRNA – Genbank accession numbers

#Accession	Sequence ID
MW827060	A_9
MW827061	A_21
MW827062	A_29
MW827063	A_167
MW827064	A_7
MW827065	A_203
MW827066	A_8
MW827067	A_75
MW827068	A_106
MW827069	A_40
MW827070	A_41
MW827071	A_119
MW827072	A_411
MW827073	A_53
MW827074	A_558
MW827075	A_180
MW827076	B_2
MW827077	B_18
MW827078	B_189
MW827079	B_32
MW827080	B_76
MW827081	B_36
MW827082	B_22
MW827083	B_14
MW827084	B_96
MW827085	B_143
MW827086	B_23
MW827087	B_118
MW827088	C_12
MW827089	C_3
MW827090	C_155
MW827091	C_15
MW827092	C_9
MW827093	C_24
MW827094	C_44
MW827095	C_19
MW827096	C_210
MW827097	C_306
MW827098	C_97

MW827099	D_7
MW827100	D_2
MW827101	D_109
MW827102	D_67
MW827103	D_15
MW827104	D_148
MW827105	D_17
MW827106	D_100
MW827107	D_49
MW827108	D_68
MW827109	D_129
MW827110	D_83
MW827111	D_59
MW827112	D_64

Table S6: Full length probes, with spacer and initiator sequences, used for HCR-FISH.

Probe Name	Initiator	Probe Sequence	Spacer	Initiator Sequence
KH 119_Eub338-1	B1	GCTGCCTCCCGTAGGAGT	ATATA	GCATTCTTTCTTGAGGAGGGCAGCAAACGGGAAGAG
KH_138_Gam42a	B3	GCCTTCCCACATCGTTT	TAAAA	AAAGTCTAATCCGTCCCTGCCTCTATATCTCCACTC
KH 133_Sacc3	B3	GCCCCCTTTCCTCCGCAG	TAAAA	AAAGTCTAATCCGTCCCTGCCTCTATATCTCCACTC
OG_Idio1	B2	TGACCAGGTGGCCGCCTT	AAAAA	AGCTCAGTCCATCCTCGTAAATCCTCATCAATCATC
OG_Mar1	B2	CCTTCCTCTACTGTACTC	AAAAA	AGCTCAGTCCATCCTCGTAAATCCTCATCAATCATC
KH 270_ OceaA167.3	B2	TAGACCCAACGGCTAGTC	AAAAA	AGCTCAGTCCATCCTCGTAAATCCTCATCAATCATC
KH 278_ OceaA29.1	B2	CTCGGATTGGCTCCACAT	AAAAA	AGCTCAGTCCATCCTCGTAAATCCTCATCAATCATC
KH 295_OceaC3.5	B2	CTCGGGTTGGCTACACCT	AAAAA	AGCTCAGTCCATCCTCGTAAATCCTCATCAATCATC
KH 288_ OceaA106_C155	B2	TCTGTGGCTAACGTCTGG	AAAAA	AGCTCAGTCCATCCTCGTAAATCCTCATCAATCATC
KH 293_ OceaA203_C155.1	B2	TCTCGGGTTGGCTCCACA	AAAAA	AGCTCAGTCCATCCTCGTAAATCCTCATCAATCATC
KH 168_Ecto8	B2	CCCGCACCCCTTCGTTTC	AAAAA	AGCTCAGTCCATCCTCGTAAATCCTCATCAATCATC
KH 171_Ecto11	B2	TTCATGAAGAGGCCCCCT	AAAAA	AGCTCAGTCCATCCTCGTAAATCCTCATCAATCATC
KH 174_Ecto14	B2	TTGGCCGCCTACGTGCCC	AAAAA	AGCTCAGTCCATCCTCGTAAATCCTCATCAATCATC
KH 297_ EctoA558.4	B2	CCGACCGCCTACGCGCAC	AAAAA	AGCTCAGTCCATCCTCGTAAATCCTCATCAATCATC
KH 285_ EctoA411.3	B2	GTTTCGGTCCAGGCAGCC	AAAAA	AGCTCAGTCCATCCTCGTAAATCCTCATCAATCATC
KH 152_Rose3	B3	ATCCGAAGATCTCGTCCG	TAAAA	AAAGTCTAATCCGTCCCTGCCTCTATATCTCCACTC
KH 154_Rhod2	B2	GCGAGTTAGCGCACCACC	AAAAA	AGCTCAGTCCATCCTCGTAAATCCTCATCAATCATC
KH 177_Cyto2	B3	CCGTTTCGGGAGCGGTCA	TAAAA	AAAGTCTAATCCGTCCCTGCCTCTATATCTCCACTC
KH 124_Brum3	B2	CAACCCGGTCATTCTGCA	AAAAA	AGCTCAGTCCATCCTCGTAAATCCTCATCAATCATC
KH 148_Spir2	B2	GCCATGATCTCTCACAGC	AAAAA	AGCTCAGTCCATCCTCGTAAATCCTCATCAATCATC

Table S7: Growth Media Recipes [6,8][50]

**AFML** (Add in the following order)	**Concentration** **mM**	**g/L**
$NaNO_3$	10	0.85
K_2HPO_4	0.3	0.05
MgSO4•7H2O	2	0.51
CaCl2•2H2O	0.3	0.046
$C_6H_8O_7$	0.18	1.8
NaCl	1000	60
KCl	22	1.7
Na_2CO_3	100	10.6
$NaHCO_3$	52	4.4
$Na_2B_4O_7$	1.3	2

- pH to 9.74 with NaOH and filter through a 0.22 µm filter under sterile conditions in a tissue culture hood.
- Add trace elements and L1 vitamins at 1:1000 right before use as previously described.

**DMSM (**pH 10 with peptone)	**mM**	**g/L**
K_2HPO_4	40	7.0
KH_2PO_4	22	3.0
$MgSO_4•7H_2O$	0.4	0.1
Peptone		1.0
Trace Element Solution		1:1000

- pH to 10 with NaOH

Table S8: Dereplicated bin set used for constructing the ribosomal protein tree

Bin	Size (Mbp)	Coverage	Completeness
NKKH001A_Ectothiorhodospiraceae_65_16	2.86	15.96	0.91
NKKH001A_Oceanospirillales_55_89	3.74	88.50	0.95
NKKH001A_Oceanospirillales_56_21	4.17	21.26	0.85
NKKH001A_Oceanospirillales_59_206	3.08	206.07	0.95
NKKH001A_Oceanospirillales_60_125	4.32	124.61	0.93
NKKH001A_Spirochaetales_59_7	1.72	7.09	0.89
NKKH001B_Bacteroidetes_40_51	4.13	50.72	0.91
NKKH001B_Bacteroidetes_44_85	3.44	84.76	0.95
NKKH001B_Bacteroidetes_53_10	3.45	9.58	0.87
NKKH001B_Ectothiorhodospiraceae_67_18	2.45	18.41	0.93
NKKH001B_Idiomarinaceae_49_171	2.82	170.77	0.93
NKKH001B_Rhodobacteraceae_59_23	4.00	23.36	0.95
NKKH001B_Rhodobacteraceae_64_110	4.66	113.54	0.95
NKKH001C_Ectothiorhodospiraceae_64_108	3.58	108.39	0.95
NKKH001C_Ectothiorhodospiraceae_64_283	2.92	283.97	0.95
NKKH001C_Flavobacteria_34_13	3.42	13.12	0.95
NKKH001C_Oceanospirillales_52_91	3.42	91.11	0.93
NKKH001C_Oceanospirillales_57_14	3.58	14.03	0.85
NKKH001C_Oceanospirillales_58_461	3.56	459.08	0.95
NKKH001C_Oceanospirillales_61_24	3.17	24.31	0.93
NKKH001D_Bacteroidetes_43_10	3.66	10.19	0.91
NKKH001D_Bacteroidetes_43_11	3.49	11.21	0.93
NKKH001D_Bacteroidetes_46_12	4.54	11.51	0.91

SUPPLEMENTARY REFERENCES

1. Richter DJ, Fozouni P, Eisen MB, King N. Gene family innovation, conservation and loss on the animal stem lineage. Elife. 2018;7. doi:10.7554/eLife.34226
2. Schiwitza S, Gutsche L, Freches E, Arndt H, Nitsche F. Extended divergence estimates and species descriptions of new craspedid choanoflagellates from the Atacama Desert, Northern Chile. Eur J Protistol. 2021;79: 125798. doi:10.1016/j.ejop.2021.125798
3. Cavalier-Smith T. Amoeboflagellates and mitochondrial cristae in eukaryote evolution: megasystematics of the new protozoan subkingdoms eozoa and neozoa. Arch fur Protistenkd. 1997;147. doi:10.1016/S0003-9365(97)80051-6
4. Nitsche F, Carr M, Hartmut A, Leadbeater BSC. Higher Level Taxonomy and Molecular Phylogenetics of the Choanoflagellatea. J Eukaryot Microbiol. 2011;58: 452–462. doi:https://doi.org/10.1111/j.1550-7408.2011.00572.x
5. King N, Young SL, Abedin M, Carr M, Leadbeater BSC. Isolation of single choanoflagellate cells from field samples and establishment of clonal cultures. Cold Spring Harb Protoc. 2009;4. doi:10.1101/pdb.prot5147
6. Booth D, Middleton H, King N. Transfection in choanoflagellates illuminates their cell biology and the ancestry of animal septins. bioRxiv. 2018; 343111.
7. King N, Young SL, Abedin M, Carr M, Leadbeater BSC. Starting and maintaining Monosiga brevicollis cultures. Cold Spring Harb Protoc. 2009;4: pdb.prot5148. doi:10.1101/pdb.prot5148
8. Joshi AA, Kanekar PP, Kelkar AS, Shouche YS, Vani AA, Borgave SB, et al. Cultivable bacterial diversity of alkaline Lonar lake, India. Microb Ecol. 2008;55: 163–172. doi:10.1007/s00248-007-9264-8
9. Atlas R. Handbook of Microbiological Media, Fourth Edition. Fourth Edi. Handbook of Microbiological Media, Fourth Edition. 2010. doi:10.1201/ebk1439804063
10. King N, Young SL, Abedin M, Carr M, Leadbeater BSC. Long-term frozen storage of choanoflagellate cultures. Cold Spring Harb Protoc. 2009;4: pdb.prot5149. doi:10.1101/pdb.prot5149
11. Levin TC, King N. Evidence for Sex and Recombination in the Choanoflagellate< i> Salpingoeca rosetta</i>. Curr Biol. 2013;23: 2176–2180.
12. Alegado RA, Brown LW, Cao S, Dermenjian RK, Zuzow R, Fairclough SR, et al. A bacterial sulfonolipid triggers multicellular development in the closest living relatives of animals. Elife. 2012;2012. doi:10.7554/eLife.00013
13. Schindelin J., Arganda-Carreras I., Frise E. Fiji is an open-source platform for biological-image analysis. Nat Methods. 2012;9: 676–682.
14. Prewitt JMS, Mendelsohn ML. The Analysis of Cell Images. Ann N Y Acad Sci. 1966;128: 1035–1053. doi:10.1111/j.1749-6632.1965.tb11715.x
15. Kuru E, Hughes HV, Brown PJ, Hall E, Tekkam S, Cava F, et al. In situ probing of newly synthesized peptidoglycan in live bacteria with fluorescent D-amino acids. Angew Chemie - Int Ed. 2012;51: 12519–12523. doi:10.1002/anie.201206749
16. Dayel MJ, Alegado RA, Fairclough SR, Levin TC, Nichols SA, McDonald K, et al. Cell differentiation and morphogenesis in the colony-forming choanoflagellate Salpingoeca rosetta. Dev Biol. 2011;357: 73–82. doi:10.1016/j.ydbio.2011.06.003
17. Laundon D, Larson BT, McDonald K, King N, Burkhardt P. The architecture of cell differentiation in choanoflagellates and sponge choanocytes. PLoS Biol. 2019;17: e3000226. doi:10.1371/journal.pbio.3000226
18. McDonald KL. Out with the old and in with the new: rapid specimen preparation procedures for electron microscopy of sectioned biological material. Protoplasma. 2014;251: 429–448. doi:10.1007/S00709-013-0575-Y
19. McDonald KL, Webb R. Freeze substitution in 3 hours or less. J Microsc. 2011;243: 227–233. doi:10.1111/J.1365-2818.2011.03526.X
20. Schindelin J, Arganda-Carreras I, Frise E, Kaynig V, Longair M, Pietzsch T, et al. Fiji: An open-

source platform for biological-image analysis. Nature Methods. Nature Publishing Group; 2012. pp. 676–682. doi:10.1038/nmeth.2019
21. Cardona A, Saalfeld S, Schindelin J, Arganda-Carreras I, Preibisch S. TrakEM2 Software for Neural Circuit Reconstruction. PLoS One. 2012;7: 38011. doi:10.1371/journal.pone.0038011
22. Peng Y, Leung HCM, Yiu SM, Chin FYL. IDBA-UD: A de novo assembler for single-cell and metagenomic sequencing data with highly uneven depth. Bioinformatics. 2012;28: 1420–1428. doi:10.1093/bioinformatics/bts174
23. West PT, Probst AJ, Grigoriev I V, Thomas BC, Banfield JF. Genome-reconstruction for eukaryotes from complex natural microbial communities. Genome Res. 2018;28: 569–580. doi:10.1101/gr.228429.117
24. Hyatt D, Locascio PF, Hauser LJ, Uberbacher EC. Gene and translation initiation site prediction in metagenomic sequences. Bioinformatics. 2012;28: 2223–2230. doi:10.1093/bioinformatics/bts429
25. Miller CS, Baker BJ, Thomas BC, Singer SW, Banfield JF. EMIRGE: Reconstruction of full-length ribosomal genes from microbial community short read sequencing data. Genome Biol. 2011;12: R44. doi:10.1186/gb-2011-12-5-r44
26. Olm MR, Brown CT, Brooks B, Banfield JF. DRep: A tool for fast and accurate genomic comparisons that enables improved genome recovery from metagenomes through de-replication. ISME J. 2017;11: 2864–2868. doi:10.1038/ismej.2017.126
27. Medlin L, Elwood HJ, Stickel S, Sogin ML. The characterization of enzymatically amplified eukaryotic 16S-like rRNA-coding regions. Gene. 1988;71: 491–499. doi:10.1016/0378-1119(88)90066-2
28. Bradley RK, Roberts A, Smoot M, Juvekar S, Do J, Dewey C, et al. Fast Statistical Alignment. Siepel A, editor. PLoS Comput Biol. 2009;5: e1000392. doi:10.1371/journal.pcbi.1000392
29. Schiwitza S, Arndt H, Nitsche F. Four new choanoflagellate species from extreme saline environments: Indication for isolation-driven speciation exemplified by highly adapted Craspedida from salt flats in the Atacama Desert (Northern Chile). Eur J Protistol. 2018;66: 86–96. doi:10.1016/j.ejop.2018.08.001
30. Carr M, Richter DJ, Fozouni P, Smith TJ, Jeuck A, Leadbeater BSC, et al. A six-gene phylogeny provides new insights into choanoflagellate evolution. Mol Phylogenet Evol. 2017;107: 166–178. doi:10.1016/j.ympev.2016.10.011
31. Rice P, Longden I, Bleasby A. EMBOSS: The European Molecular Biology Open Software Suite. Trends Genet. 2000;16: 276–277. doi:10.1016/S0168-9525(00)02024-2
32. Capella-Gutierrez S, Silla-Martinez JM, Gabaldon T. trimAl: a tool for automated alignment trimming in large-scale phylogenetic analyses. Bioinformatics. 2009;25: 1972–1973. doi:10.1093/bioinformatics/btp348
33. Stamatakis A. RAxML version 8: a tool for phylogenetic analysis and post-analysis of large phylogenies. Bioinformatics. 2014;30: 1312–1313. doi:10.1093/bioinformatics/btu033
34. Ronquist F, Huelsenbeck JP. MrBayes 3: Bayesian phylogenetic inference under mixed models. Bioinformatics. 2003;19: 1572–1574. doi:10.1093/bioinformatics/btg180
35. Hug LA, Baker BJ, Anantharaman K, Brown CT, Probst AJ, Castelle CJ, et al. A new view of the tree of life. Nat Microbiol. 2016;1: nmicrobiol201648. doi:10.1038/nmicrobiol.2016.48
36. Camacho C, Coulouris G, Avagyan V, Ma N, Papadopoulos J, Bealer K, et al. BLAST+: architecture and applications. BMC Bioinformatics. 2009;10: 421. doi:10.1186/1471-2105-10-421
37. Edgar RC. MUSCLE: multiple sequence alignment with high accuracy and high throughput. Nucleic Acids Res. 2004;32: 1792–1797. doi:10.1093/nar/gkh340
38. Miller MA, Pfeiffer W, Schwartz T. Creating the CIPRES Science Gateway for inference of large phylogenetic trees. Gatew Comput Environ Work (GCE), 2010. 2010.
39. Pruesse E, Quast C, Knittel K, Fuchs BM, Ludwig W, Peplies J, et al. SILVA: A comprehensive online resource for quality checked and aligned ribosomal RNA sequence data compatible with ARB. Nucleic Acids Res. 2007;35: 7188–7196. doi:10.1093/nar/gkm864

40. Ludwig W, Strunk O, Westram R, Richter L, Meier H, Yadhukumar A, et al. ARB: A software environment for sequence data. Nucleic Acids Res. 2004;32: 1363–1371. doi:10.1093/nar/gkh293
41. Glöckner FO, Fuchs BM, Amann R. Bacterioplankton compositions of lakes and oceans: A first comparison based on fluorescence in situ hybridization. Appl Environ Microbiol. 1999.
42. Pernthaler A, Pernthaler J, Amann R. Fluorescence in situ hybridization and catalyzed reporter deposition for the identification of marine bacteria. Appl Environ Microbiol. 2002;68: 3094–3101. doi:10.1128/AEM.68.6.3094-3101.2002
43. DePas WH, Starwalt-Lee R, Van Sambeek L, Kumar SR, Gradinaru V, Newman DK. Exposing the three-dimensional biogeography and metabolic states of pathogens in cystic fibrosis sputum via hydrogel embedding, clearing, and rRNA labeling. MBio. 2016;7: e00796----16. doi:10.1128/mBio.00796-16
44. Amann RI, Binder BJ, Olson RJ, Chisholm SW, Devereux R, Stahl DA. Combination of 16S rRNA-targeted oligonucleotide probes with flow cytometry for analyzing mixed microbial populations. Appl Environ Microbiol. 1990;56: 1919–1925. Available: http://www.ncbi.nlm.nih.gov/pubmed/2200342%0Ahttp://www.pubmedcentral.nih.gov/articlerender.fcgi?artid=PMC184531
45. Manz W, Amann R, Ludwig W, Wagner M, Schleifer KH. Phylogenetic Oligodeoxynucleotide Probes for the Major Subclasses of Proteobacteria: Problems and Solutions. Syst Appl Microbiol. 1992;15: 593–600. doi:10.1016/S0723-2020(11)80121-9
46. Hugenholtz P, Tyson GW, Blackall LL. Design and Evaluation of 16S rRNA-Targeted Oligonucleotide Probes for Fluorescence In Situ Hybridization. Gene Probes. 2002. pp. 29–42. doi:10.1385/1-59259-238-4:029
47. Choi HMT, Calvert CR, Husain N, Huss D, Barsi JC, Deverman BE, et al. Mapping a multiplexed zoo of mRNA expression. Development. 2016;143: 3632–3637. doi:10.1242/dev.140137
48. Hibberd DJ. Observations on the ultrastructure of the choanoflagellate Codosiga botrytis (Ehr.) Saville-Kent with special reference to the flagellar apparatus. J Cell Sci. 1975;17: 191–219. Available: http://jcs.biologists.org/content/17/1/191.abstract
49. Karpov SA, Coupe SJ. A revision of choanoflagellate genera Kentrosiga Schiller, 1953 and Desmarella Kent, 1880. Acta Protozool. 1998;37: 23–27.
50. Shahinpei A, Amoozegar MA, Fazeli SAS, Schumann P, Ventosa A. Salinispirillum marinum gen. nov., Sp. nov., A haloalkaliphilic bacterium in the family ‘Saccharospirillaceae’’.’ Int J Syst Evol Microbiol. 2014;64: 3610–3615. doi:10.1099/ijs.0.065144-0
